# Active Learning Configuration Interaction for Excited-State Calculations of Polycyclic Aromatic Hydrocarbons

**DOI:** 10.1021/acs.jctc.1c00769

**Published:** 2021-11-17

**Authors:** WooSeok Jeong, Carlo Alberto Gaggioli, Laura Gagliardi

**Affiliations:** † Department of Chemistry, Nanoporous Materials Genome Center, Chemical Theory Center, and Minnesota Supercomputing Institute, University of Minnesota, Minneapolis, Minnesota 55455, United States; ‡ Department of Chemistry, Pritzker School of Molecular Engineering, James Franck Institute, Chicago Center for Theoretical Chemistry, University of Chicago, Chicago, Illinois 60637, United States; § Argonne National Laboratory, Lemont, Illinois 60439, United States

## Abstract

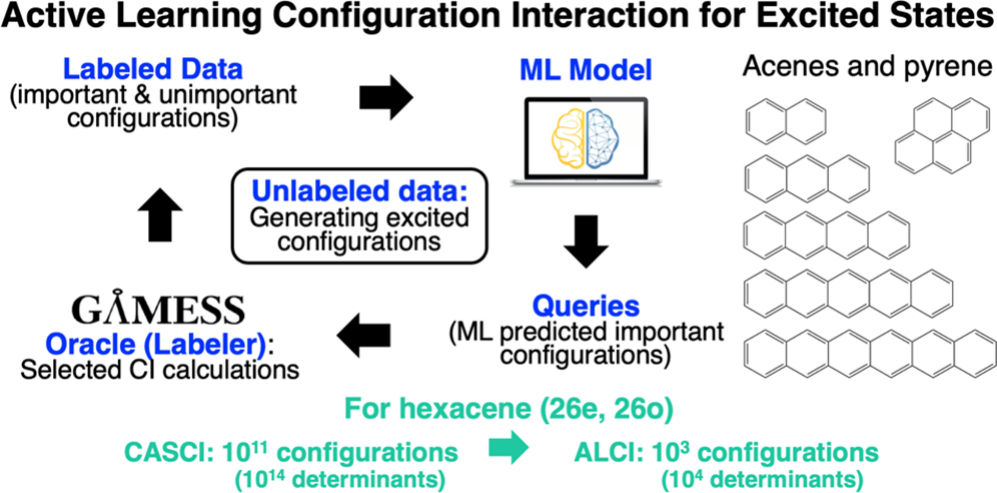

We present the active learning configuration interaction (ALCI) method for multiconfigurational calculations based on large active spaces. ALCI leverages the use of an active learning procedure to find important electronic configurations among the full configurational space generated within an active space. We tested it for the calculation of singlet–singlet excited states of acenes and pyrene using different machine learning algorithms. The ALCI method yields excitation energies within 0.2–0.3 eV from those obtained by traditional complete active-space configuration interaction (CASCI) calculations (affordable for active spaces up to 16 electrons in 16 orbitals) by including only a small fraction of the CASCI configuration space in the calculations. For larger active spaces (we tested up to 26 electrons in 26 orbitals), not affordable with traditional CI methods, ALCI captures the trends of experimental excitation energies. Overall, ALCI provides satisfactory approximations to large active-space wave functions with up to 10 orders of magnitude fewer determinants for the systems presented here. These ALCI wave functions are promising and affordable starting points for the subsequent second-order perturbation theory or pair-density functional theory calculations.

## 1 Introduction

Electronic excited states of organic materials play a key role in photovoltaics,^1−3^ light-emitting diodes,^4,5^ and photochemistry.^6−8^ The computational analysis of excited states of organic materials such as hydrocarbon molecules (e.g., aromatic molecules and polyenes)^9^ and porous organic polymers (e.g., conjugated organic polymers, hyper-cross-linked polymers, and covalent organic frameworks)^10,11^ is important to rationalize the experimental spectroscopic results and make predictions. In this regard, the most widely used methods for ab initio computations are density functional theory (DFT) and time-dependent DFT (TDDFT).^12−15^ However, a major limitation of DFT is that it may provide inaccurate results for phenomena where strong correlation plays an important role,^16^ such as bond-breaking processes,^17,18^ spin states energetics,^19−21^ and excited-state energetics.^22,23^ Strong electronic correlation, sometimes referred to as static correlation, arises when different electronic states are close in energy. Wave functions of these energetically close electronic states can be correctly described as linear combinations of several Slater determinants (SDs) or configuration state functions (CSFs) with a non-negligible contribution.^24,25^ The complete active-space self-consistent field (CASSCF) method^26^ is widely used to generate reference wave functions for strongly correlated systems. In CASSCF, an active space consisting of a given number of orbitals and electrons is chosen, and a full configuration interaction (FCI) is performed within the active space, together with the orbital optimization. Usually, the spin and spatial symmetries of the wave function are specified.

The number of SDs or CSFs scales exponentially with the size of the active space.^27^ The maximum number of electrons and orbitals that one can afford in modern computers is about 16 electronic and 16 orbitals for singlet state calculations, which corresponds to 10^8^ to 10^9^ SDs. Few examples of larger active spaces, like, for example, 22 electrons in 22 orbitals, using massive parallelization have been reported.^28^ Some approximations to reduce the number of configurations have been developed, including the restricted active-space SCF (RASSCF),^29^ the generalized active-space SCF (GASSCF),^30^ and the localized active-space SCF (LASSCF).^31^ In RASSCF and GASSCF, subspaces of electrons and orbitals are chosen, and the maximum number of electronic excitations between subspaces is restricted to the number that the user decides. In LASSCF, the active space is partitioned in multiple active subspaces, which are localized on spatially separated parts of the molecule. The FCI wave function within each subspace is obtained independently from the other active subspaces, and the total wave function is expressed as a product of these unentangled wave functions. Although these approaches allow the choice of extremely flexible active spaces, they also require an expertise in the choice of subspaces and excitation levels between different subspaces. It is also possible to only optimize the configuration interaction coefficients and not the orbital coefficients, resulting in a complete active-space configuration interaction (CASCI) calculation and the analogous GASCI and LASCI. The CASCI method, however, still involves an FCI calculation within the chosen active space.

To reduce the number of SDs or CSFs, one can perform a “selected CI (SCI)” calculation, in which many nonimportant configurations are not included in the wave function. In this case, the challenge is to identify the important configurations. Recently, SCI methods have been revisited for the computation of properties of strongly correlated systems.^32−36^ SCI methods aim to construct a compact wave function iteratively, including only a small number of determinants or configurations, to approximate the properties of the FCI wave function. One flavor of SCI is to use perturbation theory to select important configurations, like in the configuration interaction using an iterative perturbative selection (CIPSI).^37^ In the adaptive sampling CI approach,^38,39^ the single and double excitations are generated only from configurations with the highest coefficients, and then they are selected using the perturbation theory. This method has been recently employed in combination with very large active spaces, up to (52e, 52o).^40^ In the heat-bath CI (HCI) method, an approximation to the full expression of first-order perturbation is used to select configurations.^41^ In Monte Carlo configuration interaction (MCCI),^42−44^ configurations are stochastically chosen and only those with a coefficient higher than a certain threshold are retained in the wave function.

In recent years, machine learning (ML) has been increasingly used in quantum chemistry,^45−50^ to accelerate coupled-cluster calculations,^51,52^ excited-state computations^53^ and predict quantum-mechanical wave functions,^54^ to only mention a few works. In particular, an active learning (AL) approach^55^ has been used to minimize the amount of training data, thus reducing the overall training cost. In AL, the performance of a supervised ML model can be maximized with fewer labeled data if the ML model can choose data for the next training step from those learned in previous training steps. AL schemes have been successfully integrated into quantum chemistry, in combination with molecular dynamics^56−58^ and for materials discovery,^59^ especially when unlabeled data (e.g., new atomic configurations or new crystal structures) can be easily generated, while labeling of the data is difficult and time consuming (e.g., obtaining quantum-mechanical (QM) properties via ab initio calculations).

Recently, an active ML approach has been used to identify important configurations in SCI ground-state calculations of small molecules. In the method called machine learning configuration interaction (MLCI),^60^ an artificial neural network (ANN) regression model has been used to learn on the fly to choose important configurations in an iterative SCI scheme. This method significantly reduces the number of iterations to converge for the selection of configurations and requires less time compared to other approaches such as CIPSI and MCCI. MLCI recovered up to 98% of the FCI correlation energy for some multireference problems, like the computation of the dissociation of CO and H_2_O. In the following paper, MLCI was used to compute potential energy curves for N_2_, CO, and H_2_O, and the results were of FCI quality.^61^ Another example is an ML-based SCI method called Chembot, which utilizes a support vector machine (SVM) with a Gaussian radial basis function (RBF) kernel.^62^ Unlike the MLCI approach, the Chembot method adopted an SVM model to directly classify important and not important configurations to iteratively construct the wave function. By developing features using charge density matrix and configuration energy and inclusion of heuristics for better training data selection, Chembot can reach chemical accuracy and near exactness in total energy calculations for H_4_, H_2_C, and H_2_O. Both MLCI and Chembot have focused on the ground state of small molecules. In different approaches, ANNs have been employed to determine the relative weights of configurations for computing the ground state of one- and two-dimensional Heisenberg spin chains without repeated SCI calculations,^63^ and reinforcement learning techniques have been tested to calculate the ground-state energies for dissociation curves of CO, N_2_, and an H_8_ chain and larger hydrogen rings up to H_14_.^64^

**Figure 1:**
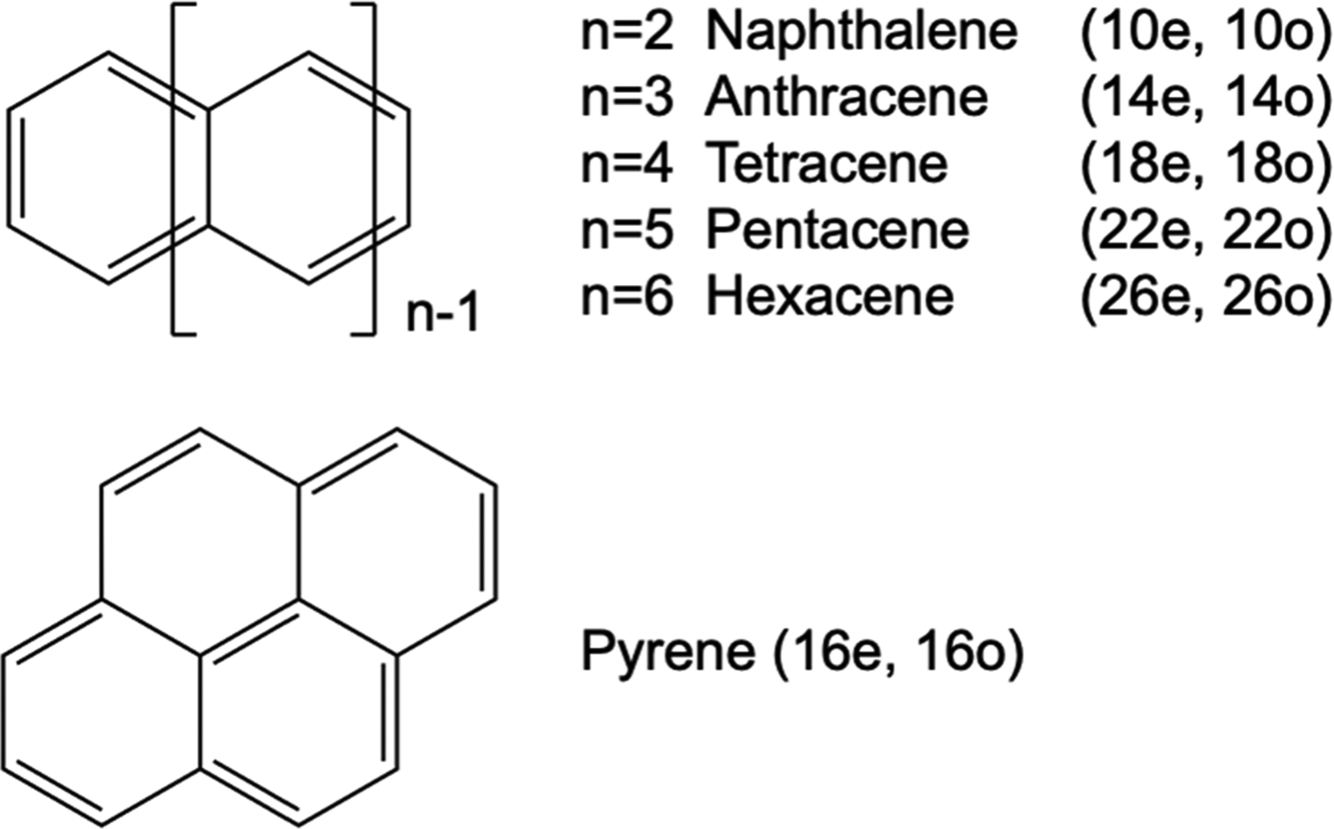
Molecular structure of polyacenes and pyrene. *n* is the number of fused benzene rings. (*x*, *y*) is the active-space size where *x* is the number of active electrons (herein, the number of π electrons) and *y* is the number of active orbitals (i.e., the number of π bonding and π* antibonding orbitals).

Inspired by the MLCI approach, we developed an active ML protocol to find important configurations to perform CASCI calculations with large active spaces. We apply the method to compute the lowest singlet–singlet excitation of several polycyclic aromatic hydrocarbons (PAH), as shown in Figure 1. We focus on linear PAH, going from naphthalene to hexacene, and on pyrene, as an example of a nonlinear PAH. These molecules are chosen because it is known that their first singlet excited state acquires significant multireference character as the size increases, resulting in interesting electronic structure and properties as promising organic optoelectronic materials.^65−67^

The paper is structured as follows: in Section 2, we describe the active learning CI (ALCI) protocol, the protocol of the quantum-mechanical calculations, and the machine learning methodologies. In Section 3, we describe the ALCI results; in Section 4, we offer our conclusions and a perspective about the use of the ALCI method.

## 2 Active Learning Configuration Interaction Protocol

To find important configurations within the full configuration space spanned by the active space, we devised an active learning CI protocol. A scheme of the protocol is provided, which focuses on the “active learning” idea first and then more details on how to implement the scheme are provided.

**Figure 2:**
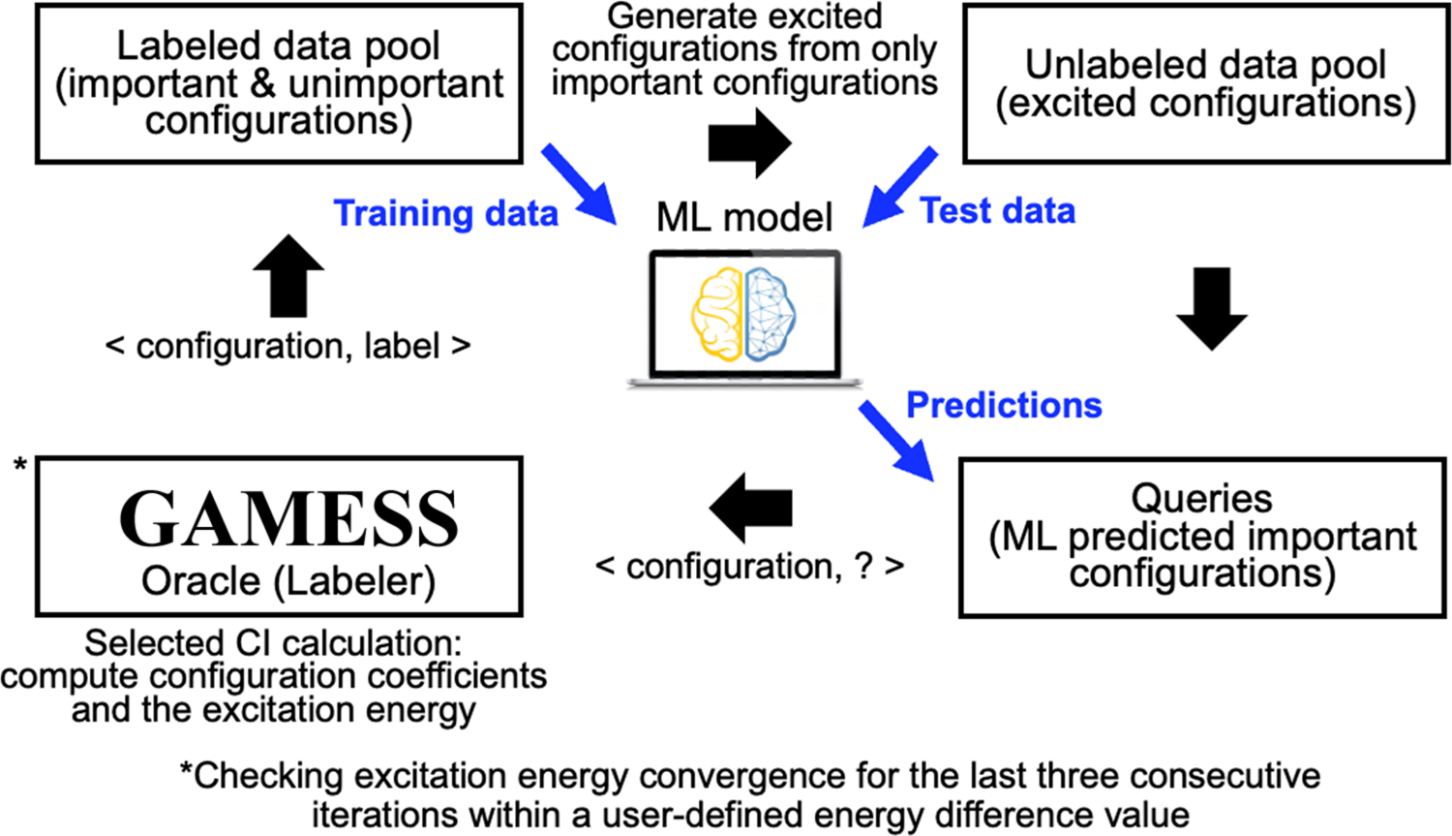
Active learning scheme for finding important configurations in iterative selected CI calculations.

As illustrated in Figure 2, our ALCI protocol uses an iterative workflow based on a pool-based active learning scheme that separates configurations into a labeled and unlabeled data pool. An oracle (i.e., the labeler that is an external general CI program, the GENCI program^68^ in the general atomic and molecular electronic structure system (GAMESS) package^69,70^) is used for performing multiconfigurational calculations with arbitrary user-specified configurations (i.e., SCI calculations) to label configurations into unimportant (label “0”) and important (label “1”), and then the configurations and their labels are saved in a labeled data pool. During the iterative procedure, a label for a configuration in the labeled data pool can be changed depending on the outcome of the SCI calculation. Unlabeled data are produced by generating excited configurations from only important configurations, which is the same approach as used in the adaptive sampling CI method^38,39^ (adopted in this work for its simplicity and proven efficiency to explore the FCI space) and then added to the unlabeled data pool after removing any duplicates of the original important configurations. A subset of data from the labeled data pool is utilized for training an ML model, and then the trained ML model is used to predict if the generated excited configurations in the unlabeled data pool are important or not. Queries (i.e., unlabeled configurations to be labeled by the oracle) are selected from the unlabeled data pool based on ML predictions. The selected queries are labeled with the use of GAMESS, i.e., we verify whether the ML predictions are accurate or not with the use of the oracle. We repeat this cycle to update data pools and generate excited configurations iteratively until the excitation energy is converged within a user-defined energy difference (0.01 eV is used in this work). This means that our approach relies on the variational principle to test the quality of our solution.

**Figure 3:**
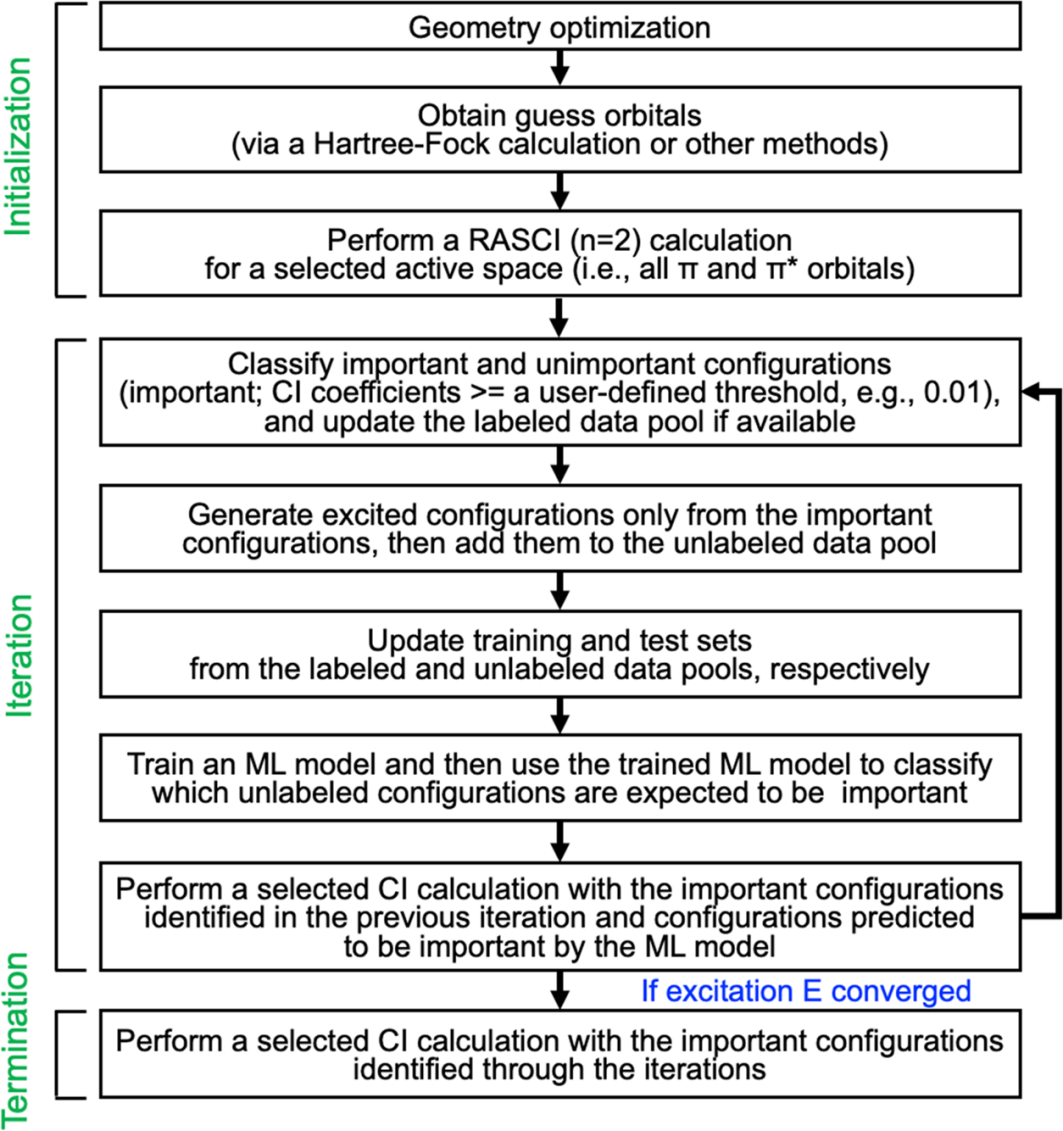
Workflow of the active learning configuration interaction (ALCI) protocol.

The detailed workflow of the active learning CI protocol controlled via an in-house Python code integrated with the GENCI program in the GAMESS package is shown in Figure 3. The protocol is divided into three steps: initialization, iteration, and termination. In the initialization step, one obtains the initial data to start the iterative process. For a given molecule, the geometry optimization is performed using the Gaussian09 software,^71^ employing the M06-L^72^ density functional and the def2-TZVP basis set.^73,74^ An ultrafine grid is used for numerical integration. We then start the ALCI protocol using the GAMESS (US) software^69,70^ with the cc-pVDZ basis set^75^ and a predefined active space (in this work, the π and π* orbitals of the acenes and pyrene). The procedure is set up to use Hartree–Fock (HF) guess orbitals, but different guesses can in principle be used. A restricted active-space CI calculation including only single and double excitations from HF (referred to as RASCI (*n* = 2)) is performed to produce the initial training data.

The second step (i.e., the iteration step) consists of the iterative scheme to identify only important configurations within the FCI space corresponding to the given active space. To start with, from the SCI calculation, configurations from outputs produced at the previous iteration (or the RASCI (*n* = 2) in the initialization step for the first iteration cycle) are extracted and then labelled either as important or unimportant based on a coefficient threshold (herein, 0.01 or 0.005). If the configuration coefficient is higher than or equal to the threshold, then the configuration is labeled as important, otherwise as unimportant. The labeled configurations are used to update the labeled data pool (i.e., add new configurations and/or update labels if labels of any existing configurations in the pool are changed). Next, for expanding the sub-CI space to search further important configurations through the following SCI calculation, excited configurations are generated up to a user-specified maximum level of excitations only from the important configurations previously identified and then added to the unlabeled data pool. After that, training and test data sets are constructed by sampling from the labeled and unlabeled data pools, respectively. Since the majority of the configurations will be unimportant, the training data set will most likely be highly imbalanced if naïve random sampling is adopted. To prevent this, a random undersampling scheme is adopted to sample the same number of unimportant configurations randomly compared to the important configurations. Noted that, however, the undersampling method is most likely going to discard a large amount of data in the majority class (i.e., unimportant configuration), resulting in a deterioration of classifier performance.^76^ This is due to the loss of data that can be important to learn the decision boundary between the minority and majority instances. Alternatively, one could utilize different approaches including oversampling,^77^ ensemble learning,^78^ and thresholding^79^ to name a few.

A binary classification machine learning model is adopted to predict whether a given configuration is important or not. For featurization of a configuration, an array that contains the active orbitals occupation numbers (either 2, 1, or 0) divided by 2 is used (see the Supporting Information, Section S1). It should be noted that the “configurations” in this work indicate molecular orbital occupation numbers, not configuration state functions (CSFs). In principle, one could utilize determinants or CSFs as features, but we adopted a simple “configuration” concept for simplicity and minimizing feature dimensions. The length of the array is therefore equal to the number of orbitals in the active space. Noteworthy, we do not have to consider the electron spin in the featurization scheme, as the SCI calculation input requires only the specification of the occupation numbers (without spin) for each configuration, and the GAMESS program then generates all possible spin combinations arising from the specified configurations. Furthermore, no symmetry of the wave function is currently used in the calculations. However, in principle symmetry could be included. Six different ML algorithms are employed to develop a binary classifier: Kernel ridge regression-based classifier (KRC),^80^ k-nearest neighbors (KNN),^81^ Gaussian processes (GP),^82^ random forest (RF),^83^ gradient boosting decision tree (eXtreme Gradient Boosting, XGBoost),^84^ and artificial neural networks (ANNs).^85^ KNN, GP, and RF classifiers are used as implemented in the scikit-learn package,^86^ while KRC is adopted by modifying the kernel ridge module in the scikit-learn package since the module supports only building a regressor model (SI, Section S2). The open-source gradient boosting Python library XGboost^84^ is used for the gradient boosting decision tree algorithm. ANN models with three hidden layers are adopted using the skorch library^87^ with PyTorch^88^ as the backend. For each iteration, hyperparameter tuning is newly performed to maximize the ML model performance (herein, the F1 score is used as a scoring method) using the HyperOpt,^89^ a Bayesian optimization Python library, with 10-fold cross-validation (CV) except for ANNs where 5-CV is used considering expensive training cost. Further details regarding the ML model training and hyperparameter tuning are available in the Supporting Information (Section S2).

The trained ML model is then employed to classify provisional important configurations from the test set that should be labeled using the GENCI program in the GAMESS package. An SCI calculation including all of the important configurations plus some (or all) important configurations predicted by the ML model (i.e., queries) is performed to update important configurations and compute the excitation energy. To reduce the computational cost, the number of the ML-predicted important configurations added for the SCI calculation is limited by a user-specified number. If the specified number is smaller than the number of training data in the previous step, the number of added ML-predicted configurations is set to the specified value, otherwise it is set to the number of training data. If the computed excitation energy is not converged, the second step is repeated until the excitation energy is converged. The calculation is converged when the excitation energy changes by less than 0.01 eV for three consecutive iterations. In the termination step, one additional SCI calculation is performed to obtain the final excitation energy value with all of the important configurations previously identified.

## 3 Results and Discussion

### 3.1 Sensitivity to Iteration Parameters

The following iteration parameters were tested to check the convergence of the ALCI calculations (in the parentheses, the baseline values for each parameter are reported): (i) maximum number of iterations for each SCI calculation (3, details are available in the Supporting Information (SI), Section S3.1), (ii) maximum sampling number of queries (2000, see the SI, Section S3.2), (iii) maximum level of excitations for each iteration (1), (iv) query sampling method (using the class probability for sampling priority), and (v) CI coefficient threshold for important configurations (0.01). Three independent calculations following the above protocol were conducted considering the stochastic nature of the ML model. For this reason, in the following, we will report the average number of iterations and average time of these three independent calculations. Naphthalene, anthracene, and tetracene were used as test systems, and a classifier based on the kernel ridge regression was employed to speed up the sensitivity test.

**Figure 4:**
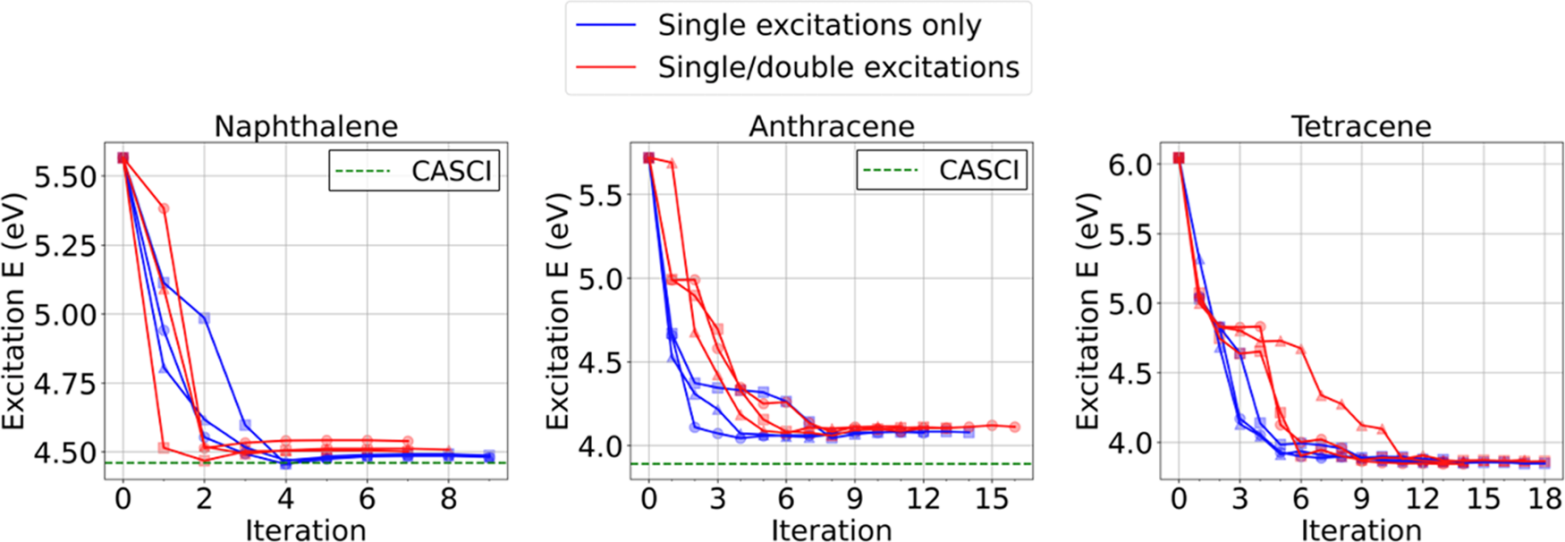
ALCI protocol convergences in terms of excitation energy depending on the maximum level of excitations for naphthalene, anthracene, and tetracene: single excitations only vs single/double excitations. Three independent protocol calculations (as indicated with different marker types) were performed for a different maximum level of excitations. Iteration zero corresponds to the RASCI (*n* = 2) calculation.

As mentioned in Section 2, unlabeled data are created as excited configurations from the important configurations identified via an SCI calculation. In this step, one has to specify the maximum level of excitation (e.g., singles, doubles, triples, or higher excitations) from the reference configurations like in multireference CI (MRCI) methods.^90^ Generating higher excitations (for example, quadruple and quintuple excitations) from a large number of reference configurations is time and memory intensive, as in the MRCI methods.^91^ Therefore, we have tested two ways: Generate (1) only single excitations and (2) single and double excitations. Note that our configuration generation method does not limit the CI expansion by truncating it to a specific excitation level like truncated CI methods, so one automatically generates higher-level excitations, as products of lower-level excitations, like in the coupled-cluster theory. As shown in Figure 4, generating both single and double excitations has no noticeable advantages over generating only single excitations. For naphthalene, including up to double excitations converges faster than including only single excitations (i.e., average 7.3 vs 9.0 iterations). However, for larger systems such as anthracene and tetracene, the higher excitations resulted in a similar or slower convergence (on average, 13.3 and 16.3 iterations, using single and double excitations, and 11.3 and 15.0 iterations, using only single excitations for anthracene and tetracene, respectively) though the converged excitation energies are similar. This suggests that single excitations from important configurations are sufficient to generate unlabeled configurations in the ALCI protocol.

**Figure 5:**
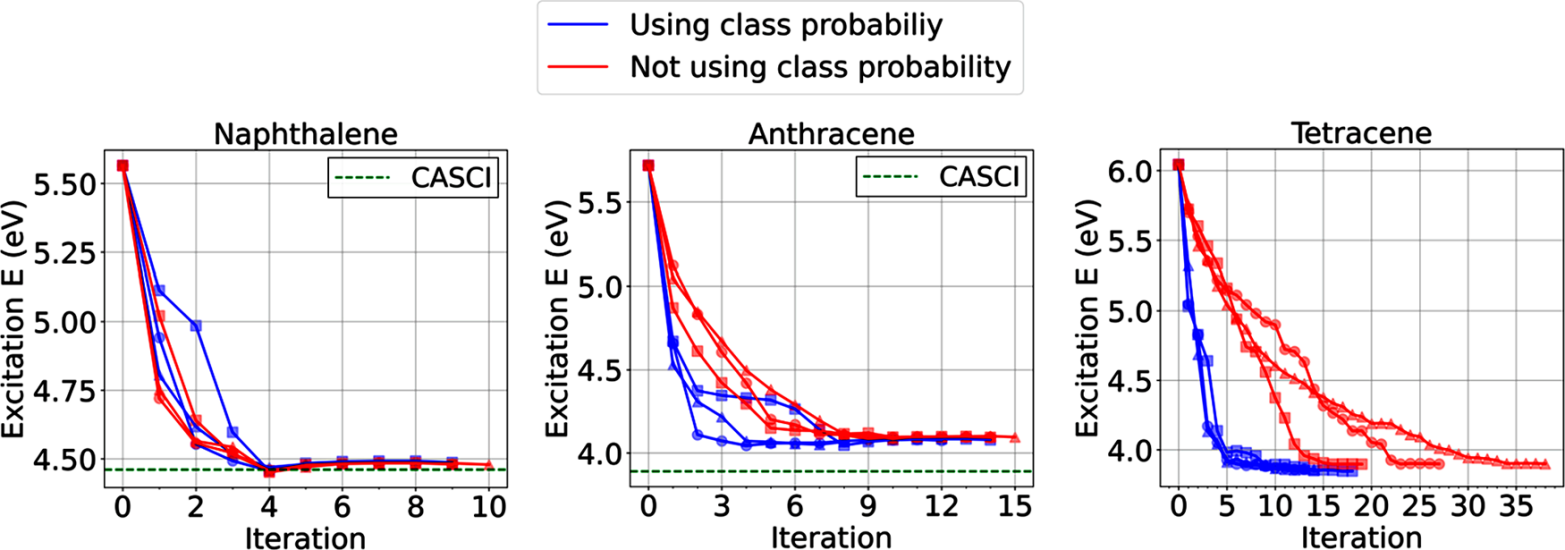
ALCI protocol convergences in terms of excitation energy depending on the use of class probability for query priority sampling for naphthalene, anthracene, and tetracene. Three independent protocol calculations (as indicated with different marker types) were performed for each case. Iteration zero corresponds to the RASCI (*n* = 2) calculation.

We implemented a unique strategy to efficiently identify important configurations, which uses class probability (i.e., the probability of each class) of an ML classifier as an uncertainty measure to decide which ML-predicted configurations should be labeled first. In general, an ML classifier predicts not only a class label (in our problem, important or unimportant) but also a probability for each class, namely, a real number from 0 to 1, with the sum of the probabilities being 1. For a binary classification problem, a class is predicted to be positive or negative if the class probability is larger or smaller, respectively, than a decision threshold, which we set to 0.5 unless otherwise specified. For example, if a class probability for a given input (e.g., an unlabeled configuration) is predicted to be larger than 0.5, then this class will be positive (e.g., important in our problem). Configurations with a high-class probability will most likely be important. It should be noted that a class probability does not correspond to a CI coefficient. The class probability shows the reliability of the ML model classification predictions (i.e., important or unimportant that is defined by a CI coefficient threshold) and so it cannot be used as an important measure for a given configuration. Figure 5 shows the comparisons of protocol calculations based on using and not using class probabilities. For naphthalene, both cases show similar convergence of the protocol calculations, resulting in the same excitation energy (4.48 eV). However, as the system size increases, sampling queries based on the class probabilities outperform random query sampling, requiring a smaller number of iterations (from 14.3 to 11.3 iterations for anthracene and from 28 to 15 iterations for tetracene on average) and producing slightly smaller excitation energies (from 4.10 to 4.07 eV for anthracene and from 3.90 to 3.86 eV for tetracene on average).

### 3.2 Comparison of Different ML Algorithms

Different ML algorithms including KRC, KNN, GP, RF, XGBoost, and ANN were tested for the ALCI protocol. Interestingly, the convergence of the excitation energy in the ALCI protocol is highly dependent on both the system size and type of the ML algorithm. For the smallest system, naphthalene, all of the ML algorithms predicted a similar excitation energy (4.48–4.50 eV). The KRC, KNN, GP, and RF classifiers took about 8–9 ALCI iterations to converge, while XGBoost and ANN required about 11 and 13 iterations, respectively (detailed ALCI results are available in the SI, Section S6.1). This difference arises since there are not enough data to train the boosting algorithm and ANN that have many model parameters and hyperparameters, as demonstrated by low ML model performance at early iterations for ANN and large fluctuations in the ML model performance for ANN and XGBoost (variations of the ML model performance, F1, can be found in the SI, Figure S10).

As the acene size increases, the performance of the different ML algorithms changes, affecting the ranking of the algorithms as far as the number of iterations is concerned (see the SI, Figure S8 and Table S7). To clearly show this, the performance of the different ML algorithms for tetracene was evaluated, in terms of both the number of iterations and computation time, as listed in Table 1. For the evaluation of the computational time, five cores (3.00 GHz Intel i9-10980XE) were used if the ML model development tools (i.e., scikit-learn and XGBoost) support parallelization of the model training and prediction (i.e., for KNN, RF, and XGBoost); otherwise, one CPU (3.00 GHz Intel i9-10980XE) core is used (for GP and KRC). For ANN, an NVIDIA Quadro RTX 8000 graphics processing unit (GPU) was used. The ANN, GP, XGBoost, and KRC algorithms require about 15 iterations, while the RF and KNN algorithms take 21 and 26 iterations, respectively. Although XGBoost and ANN require a similar number of iterations, as shown in Table 1, XGBoost is the fastest algorithm (about 3 h on average for running an ALCI full cycle), while ANN is the slowest one (about 8 h and a half on average) considering the overall computational time due to the time-consuming training procedure of ANN models even using the GPU. The KRC algorithm, which is using only one CPU core, exhibited a reasonably good performance, showing the second lowest computational cost (i.e., about 4 h and a half). Although KRC showed different convergence trends in the excitation energy calculations, most of the calculations converged to very similar excitation energies (Figure S9). Compared to the KRC, GP employed ca. 20% more time and converged to a slightly higher excitation energy. Like XGBoost, RF and KNN can also use multiple cores for parallel processing in ML model training, and these algorithms show faster convergence than XGBoost for naphthalene. However, for tetracene, RF and KNN took more iterations (i.e, 16 iterations for XGBoost vs 21 and 26 iterations for RF and KNN, respectively) and more computational time (about 3 h for XGBoost vs 4 h 40 min and 6 h 20 min for RF and KNN, respectively) than XGBoost. For all of the ML algorithms tested, a portion of the wall timing for ML predictions is negligible (up to 1.5%). Except for ANN and GP, which require 83 and 61% of the computational cost used for training ML models, the most time-consuming part of the ALCI protocol for the remaining ML algorithms is the SCI calculation (i.e., more than 90%). For the subsequent investigations, considering both the number of iterations and the computational time of the tested ML algorithms, we selected three ML algorithms, namely, ANN (smallest numbers of iterations), XGBoost (fastest algorithm for tetracene), and KRC (it uses only one CPU core but shows good performance).

**Table 1:** ALCI Protocol Results for Tetracene with Different ML Algorithms^a^

				wall time (hh:mm:ss)^b^			
ML algorithm	average number of iterations	number of important configurations	excitation energy (eV)	ML training^b^	ML predictions	SCI cal.	total^c^^,^^d^
ANN	14.8	1625	3.88	07:03:12 (83.21%)	00:00:11 (0.04%)	01:24:45 (16.66%)	08:28:34
GP	15.4	1642	3.96	03:21:24 (61.00%)	00:04:48 (1.46%)	02:03:28 (37.39%)	05:30:10
XGBoost	15.6	1753	3.87	00:07:24 (4.08%)	00:00:06 (0.05%)	02:53:15 (95.61%)	03:01:12
KRC	15.6	1749	3.90	00:25:03 (9.24%)	00:00:34 (0.21%)	04:05:05 (90.38%)	04:31:11
RF	21.2	1781	3.88	00:17:48 (6.33%)	00:00:06 (0.03%)	04:22:34 (93.39%)	04:41:09
KNN	25.7	1724	3.91	00:07:20 (1.93%)	00:01:22 (0.36%)	06:09:34 (97.51%)	06:19:01

a Results are average values of 10 independent calculations for each model that are performed to obtain better statistics.

b Wall timings measure average elapsed time for both the iteration and termination steps of the ALCI protocol, not including the initialization step (i.e., DFT optimization, HF, and RASCI (*n* = 2) calculations). To compare the computational cost, the number of CPU cores for the calculations was limited to 5 cores (Intel i9-10980XE 3.00 GHz) if ML model training/predictions can be parallelized (i.e., for KNN, RF, and XGBoost). For ANN, a GPU (NVIDIA Quadro RTX 8000) was used. GP and KRC models were trained and used with one CPU core (Intel i9-10980XE 3.00 GHz) due to the limitation of the software. SCI calculations were performed on a single CPU core due to the limitation of the GENCI program in the GAMESS package.

c Wall timing for the ML training step includes the featurization of raw data (i.e., configurations), 10-fold cross-validation for hyperparameter tuning, and retraining of an ML model with the tuned hyperparameters using all of the training data.

d Total wall time is slightly larger (25–40 s) than a sum of the ML training, ML predictions, and SCI calculations due to auxiliary processes such as transferring, saving, and loading data, etc.

### 3.3 ALCI Results for Acenes and Pyrene

#### 3.3.1 Active Spaces up to (16e, 16o)

**Figure 6:**
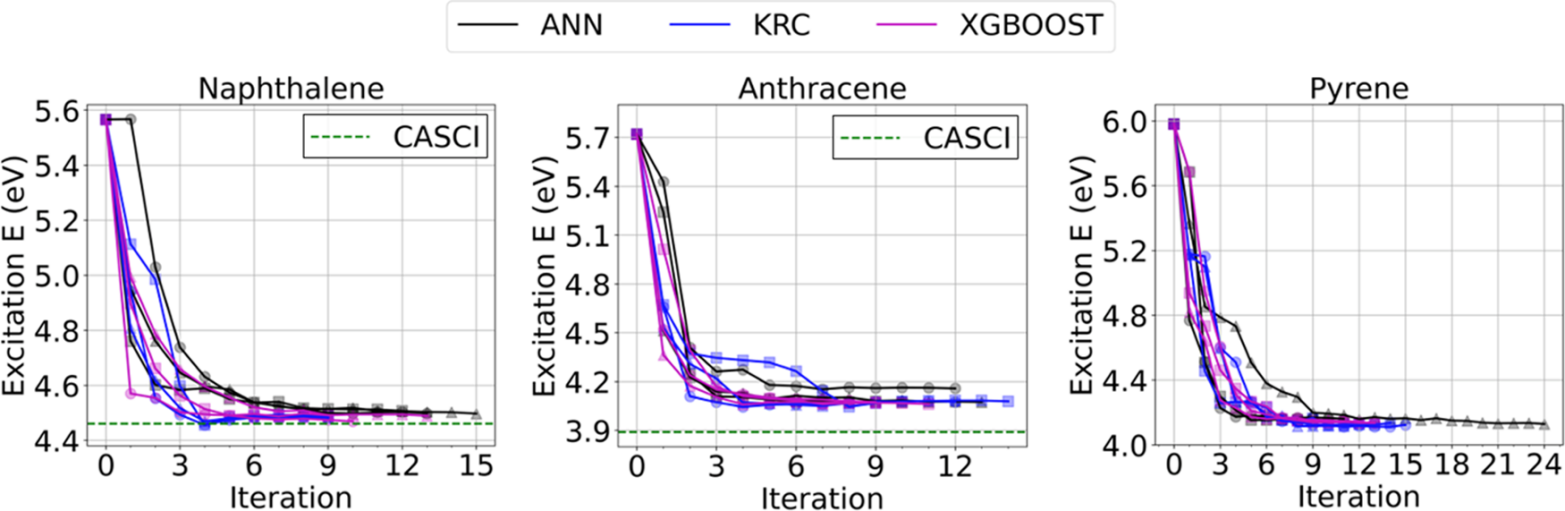
ALCI protocol convergence in terms of excitation energy for naphthalene, anthracene, and pyrene. Three independent calculations (as indicated with different marker types) are performed for each model. The CI coefficient threshold for important configuration is 0.01. Iteration zero corresponds to the RASCI (*n* = 2) calculation.

We applied the workflow presented in Figure 3 to compute the excitation energy of naphthalene. For this molecule, the π and π* active space consists of 10 electrons in 10 orbitals. The CASCI (10e, 10o) result is compared with the ALCI results (Table 2). The CASCI (10e, 10o) corresponds to 8953 configurations (63 504 SDs) and predicts an excitation energy of 4.46 eV. In Figure 6, the ALCI convergence (using ANN, KRC, and XGBoost) using a CI coefficient threshold of 0.01 is shown. At iteration 0 (i.e., RASCI (*n* = 2) calculation), the excitation energy is 5.57 eV, with about a hundred of important configurations. The excitation energy decreases in subsequent iterations, converging after 9–14 iterations. The converged excitation energies are between 4.48 and 4.50 eV. The number of important configurations in the last iteration cycle is about 360 (about 4000 SDs). It is impressive that the excitation energies are similar to the CASCI (10e, 10o) one but with 2 orders of magnitude fewer CSFs. The excitation energy decreases upon iterating, which suggests that the newly included configurations improve the excited-state wave function more significantly than the ground-state wave function. This is not surprising, since the excited state is more multiconfigurational than the ground state, and therefore, the former requires the inclusion of higher excitations for a more accurate description.

**Table 2:** ALCI Protocol Results with the Optimized Input Parameters for Naphthalene, Anthracene, and Pyrene^a^

system	ML algorithm	threshold for CI coeff.	average number of iterations	number of important configurations	number of important SDs	excitation energy (eV)
naphthalene (10e, 10o)	KRC	0.01	9.0	369	4104	4.48
		0.005	8.0	722	8379	4.45
	ANN	0.01	13.3	356	3828	4.50
		0.005	10.3	698	7942	4.46
	XGBoost	0.01	10.7	362	4072	4.48
		0.005	9.7	662	7701	4.47
	CASCI (10e, 10o)			8953^b^	63 504^b^	4.46
anthracene (14e, 14o)	KRC	0.01	11.3	1062	37 971	4.07
		0.005	11.7	2474	100 328	3.97
	ANN	0.01	11.7	923	23 462	4.10
		0.005	10.3	2353	78 577	3.98
	XGBoost	0.01	9.7	1041	37 278	4.07
		0.005	12.3	2328	97 536	4.01
	CASCI (14e, 14o)			616 227^b^	11 778 624^b^	3.89
pyrene (16e, 16o)	KRC	0.01	13.7	1444	41 961	4.13
		0.005	16.7	3660	225 039	3.98
	ANN	0.01	14.7	1243	45 457	4.15
		0.005	15.7	3424	151 508	3.99
	XGBoost	0.01	12.7	1407	40 522	4.14
		0.005	20.0	3505	155 884	4.00
	CASCI (16e, 16o)			5 196 627^b^	165 636 900^b^	3.79

a Results are average values of three separate calculations for each model that are performed to obtain better statistics.

b Total number of configurations or determinants in the active space.

Anthracene has a π–π* active space of 14 electrons in 14 orbitals. The (14e, 14o) CASCI (6.16 × 10^5^ configurations and 1.12 × 10^7^ SDs) excitation energy is 3.89 eV. The excitation energies computed with ALCI are reported in Table 2 and Figure 6. The RASCI (*n* = 2) excitation energy (at iteration = 0) is 5.72 eV, and then, it decreases during the iterative procedure, reaching the value of 4.10 eV after 10–12 iterations. With the CI coefficient threshold of 0.01, the ALCI excitation energy is about 0.2 eV higher than the CASCI value, but it corresponds to only about 10^3^ configurations (about (2–4) × 10^4^ SDs) in the last SCI calculation. So, overall in these calculations, there are 2 or 3 orders of magnitude fewer configurations/SDs than in the CASCI ones. It should be noted that using a smaller CI coefficient threshold of 0.005 results in even closer excitation energies (about 0.1 eV higher) to the CASCI limit.

The pyrene molecule is a nonlinear PAH and its active space includes 16 electrons in 16 orbitals. The CASCI (16e, 16o) corresponding to 5.20 × 10^6^ configurations (1.66 × 10^8^ SDs) predicts an excitation energy of 3.79 eV. In Figure 6, the excitation energies obtained with ALCI are shown. The RASCI (*n* = 2) excitation energy is 5.98 eV, and the iterative protocol converges to about 4.0–4.15 eV in 13–20 iterations. By using the 0.01 threshold, the ALCI method provides an excitation energy within 0.3 eV from CASCI, with the inclusion of only (1.2–1.4) × 10^3^ configurations ((4–4.5) × 10^4^ SDs), so 3 or 4 orders of magnitudes fewer than CASCI. Using a smaller threshold for the CI coefficients, 0.005, the predicted excitation energies are about 3.9 eV (i.e., a decrease of 0.1–0.16 eV compared to using the threshold of 0.01), which are closer to the CASCI result. This suggests that by adopting the lower threshold, the ALCI protocol can detect more configurations with small but non-negligible coefficients. However, in return for accuracy, a larger number of iterations and important configurations is needed in the ALCI calculations, resulting in the increased computational cost (2.2–4.7 times depending on ML algorithms. See S6.3 for more details). It is observed that all of the tested ML algorithms such as KRC, XGboost, and ANN yield similar excitation energies.

In terms of the number of screened important configurations at each iteration, Figure S11 shows that, for KRC and XGBoost, the number of important configurations increases rapidly at initial iterations (up to 3–6 iterations). In the following iterations, the increase in the configurations becomes smaller, ended in almost no increment in the last iterations. Compared to the KRC and XGBoost models, the ANN models exhibit poor reproducibility of the ALCI protocol calculations, suggesting that the hyperparameter tuning and training of the ANN models have not been totally successful. This may be due to an insufficient amount of training data for the large hyper/model parameter space of the ANN models.

Regarding timings, ALCI can be performed in about 22 min (using XGBoost with five Intel i9-10980XE @3.00 GHz) with respect to about 3 h (using one Intel i9-10980XE @3.00 GHz) required for CASCI (14e, 14o) for anthracene. For pyrene, ALCI requires only about 46 min (using XGBoost with five Intel i9-10980XE @3.00 GHz), while CASCI requires more than 91 h (using one Intel i9-10980XE @3.00 GHz). For larger active spaces, CASCI calculations become infeasible, while ALCI calculations are affordable. From the above ALCI results for naphthalene, anthracene, and pyrene, it is shown that the ALCI method is able to identify the important configurations needed to be included in the SCI calculations in few iteration cycles.

The ground- and excited-state wave functions for anthracene and pyrene have then been analyzed by comparing the active orbital occupation numbers obtained with ALCI and CASCI. These values are reported in Section S6. The differences in occupation numbers between ALCI and CASCI are within 0.03, pointing to accurate ALCI wave functions.

#### 3.3.2 Active Spaces beyond (16e, 16o)

**Figure 7:**
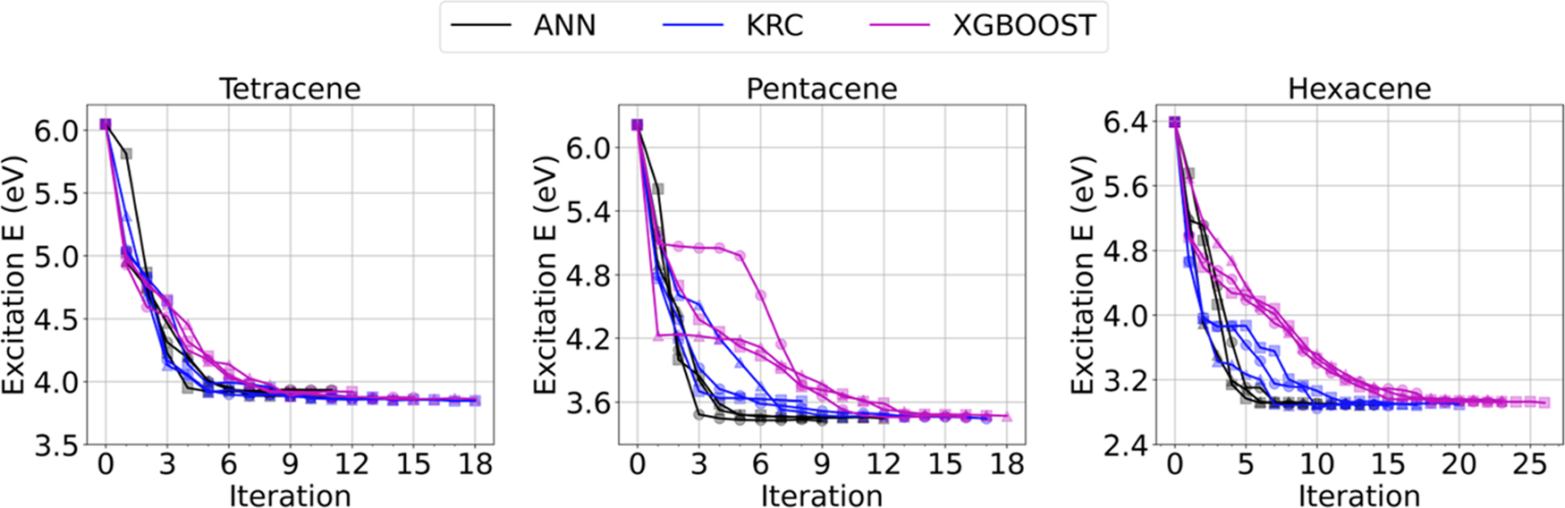
ALCI protocol convergence in terms of excitation energy for tetracene, pentacene, and hexacene. Three separate calculations (as indicated with different marker types) are performed for each model. The CI coefficient threshold for important configuration is 0.01. The iteration zero corresponds to the RASCI (*n* = 2) calculation.

We used the ALCI method to compute excitation energies for larger active spaces, for which the respective CASCI calculations are not affordable, and the results are reported in Table 3 and Figure 7. Tetracene, pentacene, and hexacene have been investigated, with active spaces of (18e, 18o), (22e, 22o), and (26e, 26o), respectively. While CASCI calculations are not feasible, experimental data are available, and therefore, they have been used as benchmarks for the ALCI results.

**Table 3:** ALCI Protocol Results with the Optimized Input Parameters for Tetracene, Pentacene, and Hexacene^a^

system	ML algorithm	threshold for CI coeff.	average number of iterations	number of important configurations	number of important SDs	excitation energy (eV)
tetracene (18e, 18o)	KRC	0.01	15.0	1759	53 213	3.86
		0.005	22.7	4788	251 320	3.74
	ANN	0.01	10.3	1491	32 663	3.91
		0.005	18.0	3944	179 952	3.73
	XGBoost	0.01	15.0	1741	53 125	3.88
		0.005	24.3	4642	234 622	3.75
	CASCI (18e, 18o)			44 152 809^b^	2 363 904 400^b^	N/A
pentacene (22e, 22o)	KRC	0.01	12.7	1793	31 491	3.50
		0.005	25.0	4780	216 678	3.46
	ANN	0.01	10.7	1713	22 622	3.44
		0.005	16.0	4195	175 336	3.48
	XGBoost	0.01	17.0	1979	47 645	3.47
		0.005	25.0	4941	231 838	3.46
	CASCI (22e, 22o)			3 241 135 527^b^	497 634 306 624^b^	N/A
hexacene (26e, 26o)	KRC	0.01	17.3	2430	27 468	2.89
		0.005	18.7	4061	58 237	3.02
	ANN	0.01	12.3	2366	24 760	2.89
		0.005	15.0	4574	101 015	3.03
	XGBoost	0.01	24.0	2670	51 247	2.93
		0.005	36.3	5952	245 534	3.03
	CASCI (26e, 26o)			241 813 226 151^b^	108 172 480 360 000^b^	N/A

a Results are average values of three separate calculations for each model that are performed to obtain better statistics.

b Total number of configurations or determinants in the active space.

For tetracene, ALCI with a CI coefficient threshold of 0.01 yields an excitation energy of about 3.9 eV in 10–15 cycles, including about (1.5–1.7) × 10^3^ configurations ((3–5) × 10^4^ SDs). A decrease of about 0.1–0.2 eV in the predicted excitation energy is achievable by decreasing the CI coefficient threshold to 0.005 at the expense of computation cost (i.e., 6–9.4 times). For pentacene, ALCI converges to an excitation energy of about 3.4–3.5 eV in 11–17 iterations, including about (1.7–2) × 10^3^ configurations ((2–3) × 10^4^ determinants) in the wave function when the 0.01 threshold is chosen. Finally, in the case of hexacene, ALCI converges to an excitation energy of about 2.9 eV in 12–24 iterations, including only about (2.4–2.7) × 10^3^ configurations ((2.5–5.1) × 10^4^ SDs) in the wave function. Considering that the number of determinants for a CASCI (26e, 26o) calculation is about 1.08 × 10^14^, ALCI can reduce this number by 10 orders of magnitude, making this calculation feasible with a reasonable computational time (about 19 h using KRC and ANN and 50 h with XGBoost. See the SI, Section S6.6). Unexpectedly, we see that excitation energies using ALCI with the lower threshold of 0.005 showed almost no improvement for pentacene and even deterioration for hexacene. This could happen if less relevant configurations (i.e, configurations that only marginally contribute to the overall energy) from the vast CASCI configuration space are included in the training set for each iteration by lowering the CI coefficient threshold for such a large system. To overcome this issue for larger systems, different featurization methods and advanced ranking strategies for choosing queries need to be developed and integrated with our ALCI protocol in future work.

With increasing acene size, the computational time for the ML model training/predictions becomes negligibly small while the computational cost for the SCI calculations grows especially when using KRC or XGBoost (detailed timing data for arbitrary selected ALCI calculations are reported the SI, Section S6.7). For example, the relative computational costs for the ML model training/predictions and SCI calculations for hexacene are 3.2 and 96.6%, respectively, when using KRC, and 0.8 and 99.0%, respectively, when using XGBoost. In the case of ANN, although the time of the SCI calculations increases with the acene size, for hexacene, about half of the time is spent in the ANN model training (i.e., 48.7%). Regarding the overall computational cost for the ALCI protocol, XGBoost, which is the fastest algorithm for tetracene, becomes the slowest one among the three ML algorithms tested for pentacene and hexacene (about 11 and 50 h for pentacene and hexacene, respectively, on average using five Intel i9-10980XE @3.00 GHz. See the SI, S6.5 and S6.6). The slow convergence of the ALCI calculations based on XGBoost for the large systems (i.e., pentacene and hexacene) results from the fact that, as shown in Figure S11, XGBoost cannot identify important configurations effectively as the iterations proceed compared to the KRC and ANN algorithms, resulting in larger (average) numbers of important configurations (i.e., in the case of hexacene, 2670 for XGBoost vs ca. 2400 for KRC and ANN) and iterations (about 24, 17, and 12 iterations for XGBoost, KRC, and ANN, respectively) than other ML algorithms. On the other hand, ANN performs better as the acene size increases, leading to a similar computational cost of about 19 h compared to the fastest ML algorithm, KRC (see the Supporting Information, Table S14). This trend indicates that ANN would be the best-performing ML algorithm in the ALCI method for systems with active spaces larger than (26e, 26o).

#### 3.3.3 Comparison with Experimental Data

**Figure 8:**
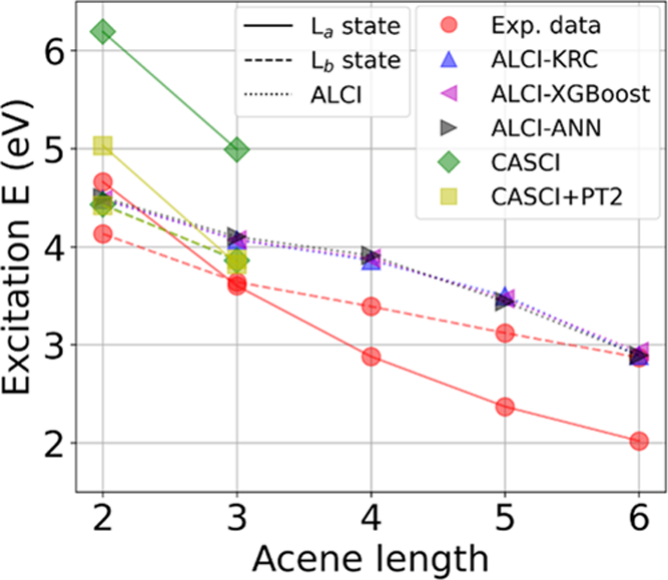
Excitation energy for different acene lengths computed with ALCI (using KRC, XGBoost, and ANN as ML algorithms) and CASCI. The experimental results from ref (92) are also reported.

Finally, the excitation energies using ALCI (with KRC, XGBoost, and ANN as ML algorithms) and CASCI are compared to experimental values in Figure 8.^92^ Before discussing our results, it should be noted that a direct comparison between experimental and computed excitation energies is often difficult because experimentally one measures band maxima, which are usually red-shifted with respect to the computed vertical excitations.^93^ For all of the acenes analyzed in this work, there are two possible lowest singlet excited states depending on the orbitals that take part in the excitation process. One of them is labeled L_a_, corresponding to a HOMO to LUMO excitation, while the other (L_b_) arises from a mixture of the HOMO – 1 to LUMO and the HOMO to LUMO + 1 excitations.^67,92,94^ The computed lowest excited states with different methods (along with the experimental ones) are shown in Table 4. Note that the ALCI method yields almost the same excitation energy using the three different ML algorithms. The ALCI and CASCI lowest excited state is L_b_ for both naphthalene and anthracene. The CASCI method recovers only part of the electron correlation, and therefore, a subsequent calculation on top of the CASCI wave function is required to compute accurate energies. This is usually done using the perturbation theory (PT2)^95,96^ or the more recent pair-density functional theory (PDFT)^97^ starting from a multiconfigurational wave function. We thus performed a PT2 calculation on top of CASCI, CASCI + PT2, to compute excitation energies using the computational procedure reported in Section S8. The lowest excited state for anthracene is now L_a_, as experimentally found. Moreover, we notice that the PT2 correction does not change sizably the L_b_ excitation energy, but it lowers the L_a_ energy by about 1 eV, with respect to CASCI. ALCI and CASCI predict the same excitation energy for naphthalene, while ALCI yields a higher excitation energy than CASCI (by about 0.2 eV) for anthracene. For larger acenes, CASCI calculations are not affordable, and therefore, we compare the ALCI results only with experiments. For the series of acenes analyzed, the computed excitation energy decreases with the acene length, reproducing the experimental trend. The ALCI excitation energies overestimate the experimental ones by about 0.3 eV when L_b_ is the lowest excited state. The ALCI and experimental value discrepancy is higher (about 0.8 eV) when L_a_ is the lowest excited state. We notice that the ALCI excitation energies of L_a_ states are higher with respect to the experimental ones, but the PT2 correction would lower them, based on the naphthalene and anthracene CASCI + PT2 results, and therefore, this correction will be needed to achieve higher accuracy. The important finding is that the ALCI results can reproduce the experimental trend, and since the ALCI wave functions are similar to the CASCI ones, but they are considerably less expensive, (Section S7), they can be used as starting points for subsequent PT2 or PDFT calculations.

**Table 4:** First Singlet Vertical Excited States for Acenes Determined by Different Methods

	naphthalene	anthracene	tetracene	pentacene	hexacene
exp. data	L_b_	L_a_	L_a_	L_a_	L_a_
CASCI	L_b_	L_b_	N/A	N/A	N/A
CASCI + PT2	L_b_	L_a_	N/A	N/A	N/A
ALCI	L_b_	L_b_	L_b_	L_a_	L_a_

## 4 Conclusions

We developed an iterative active machine learning method, called the active learning configuration interaction (ALCI) method, that can be used to efficiently identify important configurations in large active spaces calculations. As a first application, we tested the ALCI method to compute the first singlet excited state of naphthalene, anthracene, tetracene, pentacene, hexacene, and pyrene. ALCI can identify the most important configurations within 10–20 iterations, yielding excitation energies that differ at most by 0.3 eV from the CASCI ones but with orders of magnitude fewer configurations. We employed ALCI to calculate excitation energies for active spaces up to (26e, 26o), for which CASCI is unfeasible. In the cases where we could not perform the CASCI calculation, we compared ALCI excitation energies to the experimental ones and found that ALCI is able to reproduce the experimental trend, namely, the lowering of the excitation energy with the increasing acene length. For hexacene (26e, 26o), ALCI converges with only about 2400 configurations (25 000 Slater determinants) in the wave function, with a reduction of the number of determinants of about 10 orders of magnitude with respect to the corresponding CASCI. This study shows that, among the various ML algorithms tested, namely, KRC, KNN, GP, RF, XGBoost, and ANN, the ANN model exhibits the best ALCI performance in terms of both fewer iterations and the lowest computational cost of about 19 h for the largest system, hexacene. Integrating the ALCI protocol with recently developed efficient CI solving algorithms^40,98−100^ could enable us to investigate even larger active spaces. Finally, the ALCI wave functions can be used as the starting point for PT2 or PDFT subsequent calculations to achieve higher accuracy in predicting excitation energies.

## Supporting Information Available

Additional details and data on the extraction and featurization of raw data; hyperparameter tuning; sensitivity analysis of iteration parameters; generation method of excited configurations from a given reference configuration; ML model performance; ALCI protocol results; comparison of ALCI and CASCI wave functions; computational methods for the CASCI + PT2 calculations (PDF)
GAMESS input files used to perform Hartree–Fock, CASCI, and RASCI (*n* = 2) calculations for all of the polycyclic aromatic hydrocarbons studied in this work (ZIP)
