# Supplementary material for: Active Learning Configuration Interaction for Excited-State Calculations of Polycyclic Aromatic Hydrocarbons
Source: J Chem Theory Comput. 2021 Nov 17;17(12):7518–30. doi: 10.1021/acs.jctc.1c00769 (PMC8675132; doi:10.1021/acs.jctc.1c00769)
Supplement: Supplementary file 1 — ct1c00769_si_001.pdf [file ct1c00769_si_001.pdf]

Supporting Information:

Active Learning Configuration Interaction for Excited State Calculations of Polycyclic Aromatic Hydrocarbons

WooSeok Jeong[a†], Carlo Alberto Gaggioli[b†] and Laura Gagliardi[b,c*]

a) Department of Chemistry, Nanoporous Materials Genome Center, Chemical Theory Center, and Minnesota Supercomputing Institute, University of Minnesota, Minneapolis, Minnesota 55455, United States; Present Address: Center for AI and Natural Sciences, Korea Institute for Advanced Study, Seoul 02455, Republic of Korea

b) Department of Chemistry, Pritzker School of Molecular Engineering, James Franck Institute, Chicago Center for Theoretical Chemistry, University of Chicago, Chicago, Illinois 60637, United States

c) Argonne National Laboratory, Lemont, Illinois 60439, United States

†W.J. and C.A.G. contributed equally to this work

*To whom correspondence should be addressed.

Table of Contents

S1. Raw Data Extraction and Featurization……………………………………………………...S2
S2. Hyperparameter Tuning and ML Model Training……………………………………………...S3
S3. Sensitivity to Iteration Parameters……...…………………………………………………...S7
S4. Generation of Excited Configurations...……………………………………………………S15
S5. Machine Learning Model Performance……………………………………………………S16
S6. Active Learning CI Protocol Results…..……………………………………………………S19
S7. Comparison of ALCI and CASCI results……………...……………………...……………..S29
S8. Computational methods for the CASCI+PT2 calculations……………………………...…S31

References……………………………………………………………………..……………..S32

S1. Raw Data Extraction and Featurization

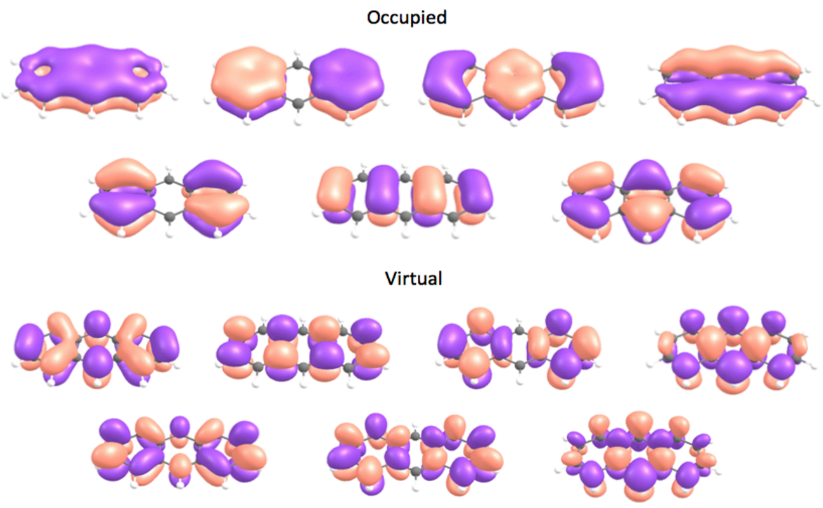

Figure S1. Anthracene HF π (occupied) and π * (virtual) orbitals chosen for the anthracene active space.

To featurize configurations to train an ML model, configurations (i.e., molecular orbital occupation numbers) and corresponding CI coefficients (to be exact, a sum of CI coefficients for determinants of a same configuration) are extracted from GAMESS(US)[1,2] output files first. Since occupation numbers are recorded separately for alpha and beta orbitals in the output files (i.e., as a form of determinants as shown in Figure S2), the first step is merging the occupation numbers to obtain configurations for each orbital. Noted that, in principle, one can utilize determinants or configuration state functions (CSFs) as features. However, we adopted the simpler concept of spatial orbital occupation numbers, configurations, for simplicity and also for minimizing feature dimensions. Configurations are extracted for both the ground and lowest singlet excited states since we compute the first singlet excitation energy. For the same configuration, CI coefficients of the ground and excited states are compared, and then the largest CI coefficient between them is only saved to consider important configurations in terms of both ground and excited states. The sign of the CI coefficients is not considered since only the absolute values of the CI coefficients are compared to determine which configurations are important. At the last step, each occupation numbers of the orbitals in each configurations are separated and divided by 2, and then saved in separated columns as a format of pandas[3] dataframe for easy handling of the features to train an ML model. Based on

a prespecified threshold for CI coefficients (herein, we used 0.01 or 0.005), configurations are classified as important and unimportant, and labeled as “1” or “0”, respectively.

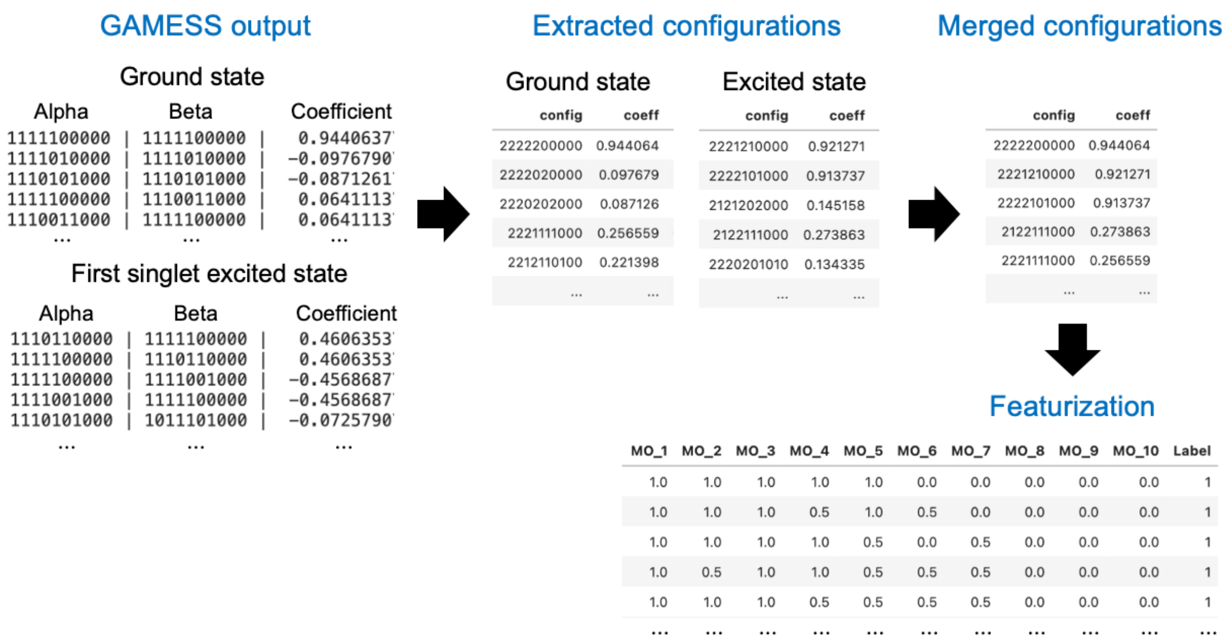

Figure S2. Data extraction and featurization procedure of configurations.

S2. Hyperparameter Tuning and ML Model Training

To develop kernel ridge regression-based classifier (KRC), k-nearest neighbor (KNN), Gaussian processes (GP), and random forest (RF) classifiers, the scikit-learn[4] (ver. 0.23.2), an open-source machine learning Python library, is used. For the gradient boosting decision tree model, XGBoost[5] (ver 1.2.0) was adopted. Regarding ANNs, a typical feed-forward fully-connected neural network with three hidden layers is developed using the skorch[6] Python library (ver. 0.9.1.dev0) with PyTorch[7] (ver. 1.7.0) as the backend. Due to the absence of a kernel ridge-based classifier model in the scikit-learn, the KRC model is developed by modifying the existing kernel ridge regression (KRR) model in the scikit-learn. To do this, predictions of the KRR model (i.e., to predict an absolute value of CI coefficient for a given configuration) are transformed to prediction probabilities first by using the min-max scaling. After that, final class predictions (i.e., important or unimportant) are made based on a threshold of 0.5 (50%). If a prediction probability is larger than 0.5 then class prediction is important, otherwise unimportant.

$$prediction\ probability = \frac{KRR\ prediction - \min(KRR\ predictions)}{\max(KRR\ predictions) - \min(KRR\ predictions)}$$

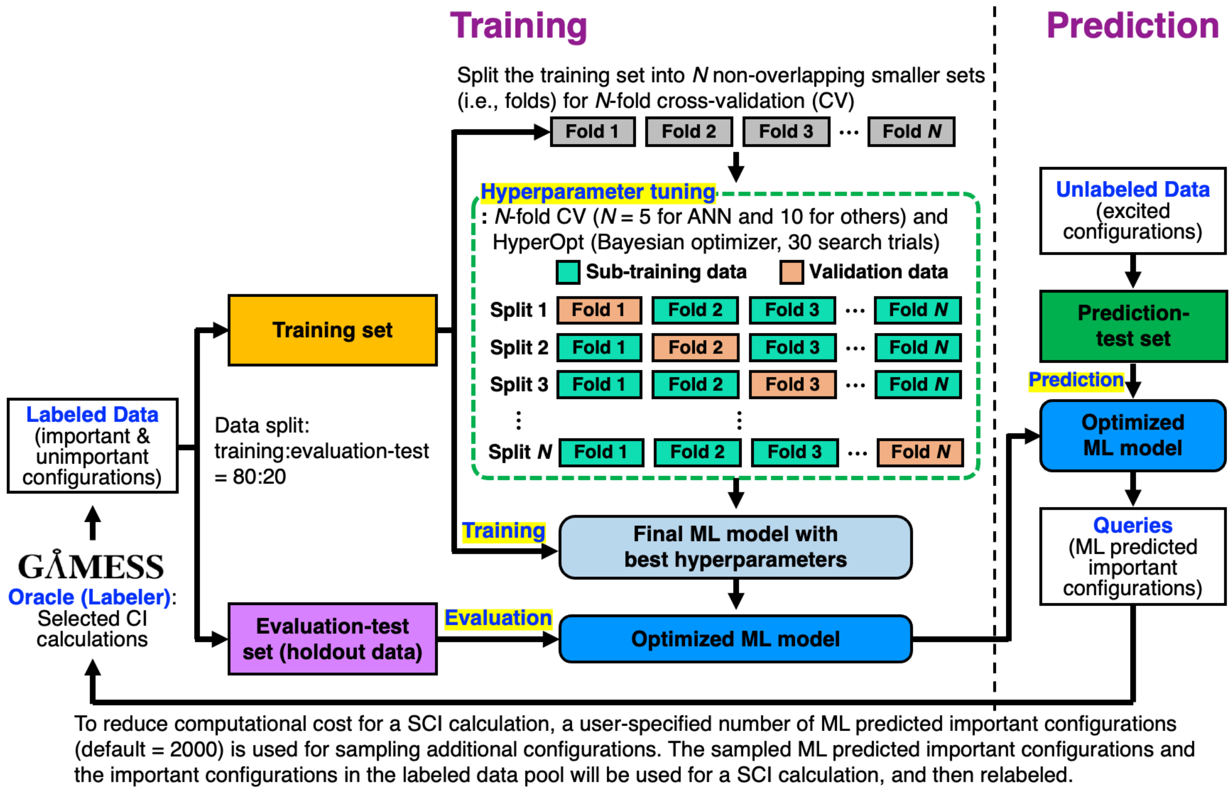

Figure S3. Workflow of the supervised ML model development in the ALCI protocol.

Hyperparameter tuning is performed at each iteration to maximize the ML model performance since the training data set is updated at every iteration in the ALCI protocol. The F1scoring metric is used for both hyperparameter tuning and evaluation of ML models. A Bayesian optimizer, HyperOpt,[8] is used to search the best hyperparameters from a given hyperparameter search space as listed in Table S1. A 10-fold cross-validation (CV) and 30 search trials are utilized except for ANNs where 5-CV and 20 search trials are used due to the higher computational cost compared to other ML algorithms. Once the best hyperparameters are determined, 80% of training data are used to retrain a ML model with the identified hyperparameters to maximize the ML performance, and the remaining 20% of training data are used to compute the ML performance (i.e., as a validation data set). For ANNs, early-stopping is adopted to avoid an over-fitting

problem. Figure S3 illustrates the overall workflow of the ML model procedure within the ALCI protocol. There are two different test sets unlike in conventional ML-model development procedures with CV: evaluation-test and prediction-test sets. Since we only check real labels for a part of the ML-predicted important configurations (i.e., we sample up to 2000 configurations to reduce the SCI calculation cost) via a SCI calculation, we do not know whether other unlabeled configurations are important or not. We thus cannot evaluate the performance of the ML model used in the "Prediction" stage. One may try to run SCI calculations with all the unlabeled configurations, but the computational cost increases, and this would become intractable for systems large than anthracene. Instead, we evaluate the ML-model performance at the "Prediction" stage since we have configurations and their labels together in the evaluation-test set. These computed ML model performance values are reported in Figure S10.

Table S1. Hyperparameter search space.

<table>
<tr><th>algorithm</th><th>hyperparameter</th><th>search space</th></tr>
<tr><td rowspan="3">KRR</td><td>kernel type</td><td>linear, radial basis function (RBF), Laplacian</td></tr>
<tr><td>regularization strength (α)</td><td rowspan="2">1e-6, 1e-5, 1e-4, …, 1e1, 1e2, 1e3, 1e4,
2e-6, 2e-5, 2e-4, …, 2e1, 2e2, 2e3, 2e4,
…
8e-6, 8e-5, 8e-4, …, 8e1, 8e2, 8e3, 8e4,
9e-6, 9e-5, 9e-4, …, 9e1, 9e2, 9e3, 9e4</td></tr>
<tr><td>γ (for RBF and Laplacian)</td></tr>
<tr><td rowspan="4">KNN</td><td>n_neighbor</td><td>1, 2, 3, 4, …, 28, 29</td></tr>
<tr><td>weights</td><td>uniform, distance</td></tr>
<tr><td>leaf_size</td><td>1, 2, 3, 4, …, 198, 199</td></tr>
<tr><td>algorithm</td><td>auto, ball_tree, kd_tree, brute</td></tr>
<tr><td rowspan="4">GP</td><td>kernel type</td><td>RBF, Matern, RationalQuadratic</td></tr>
<tr><td>length scale</td><td>1e-6, 1e-5, 1e-4, …, 1e1, 1e2, 1e3, 1e4,
2e-6, 2e-5, 2e-4, …, 2e1, 2e2, 2e3, 2e4,
…
8e-6, 8e-5, 8e-4, …, 8e1, 8e2, 8e3, 8e4,
9e-6, 9e-5, 9e-4, …, 9e1, 9e2, 9e3, 9e4</td></tr>
<tr><td>ν (for Matern)</td><td>0.5, 1.5, 2.5</td></tr>
<tr><td>α (for RationalQuadratic)</td><td>1e-6, 1e-5, 1e-4, …, 1e1, 1e2, 1e3, 1e4,
2e-6, 2e-5, 2e-4, …, 2e1, 2e2, 2e3, 2e4,
…
8e-6, 8e-5, 8e-4, …, 8e1, 8e2, 8e3, 8e4,
9e-6, 9e-5, 9e-4, …, 9e1, 9e2, 9e3, 9e4</td></tr>
</table>

algorithm	hyperparameter	search space
RF	n_estimators	10, 11, 12, …, 198, 199
	max_depth	None, 3, 4, 5, …, 28, 29
	max_features	auto, log2, None | 0.5, 1, 0.1
	max_leaf_nodes	None, 10, 11, 12, …, 98, 99
	min_samples_split	2, 5, 10
XGBoost	n_estimators	10, 11, 12, …, 198, 199
	max_depth	None, 3, 4, 5, …, 28, 29
	learning_rate	1e-6, 1e-5, 1e-4, 1e-3, 1e-2, 1e-1,<br>2e-6, 2e-5, 2e-4, 2e-3, 2e-2, 2e-1,<br>…<br>8e-6, 8e-5, 8e-4, 8e-3, 8e-2, 8e-1,<br>9e-6, 9e-5, 9e-4, 9e-3, 9e-2, 9e-1, 1e0
	min_child_weight	0.1, 0.5, 1, 2, 3, …, 8, 9, 10
	subsample	0.5, 0.6, 0.7, 0.8, 0.9, 1
	colsample_bytree	0.4, 0.6, 0.8, 1
	colsample_bylevel	0.4, 0.6, 0.8, 1
	reg_alpha	0.01, 0.03, 0.05, 0.1, 0.3
	reg_lambda	0.01, 0.03, 0.05, 0.1, 0.3
	importance_type	gain, weight, cover, total_gain, total_cover
ANN	number of hidden layers	3
	number of neurons per hidden layer	[number of active orbitals/2, number of active orbitals*2] with a spacing of (number of active orbitals/2)
	learning_rate	1e-6, 1e-5, 1e-4, 1e-3, 1e-2, 1e-1,<br>2e-6, 2e-5, 2e-4, 2e-3, 2e-2, 2e-1,<br>…<br>8e-6, 8e-5, 8e-4, 8e-3, 8e-2, 8e-1,<br>9e-6, 9e-5, 9e-4, 9e-3, 9e-2, 9e-1, 1e0
	optimizer	SGD, RMSprop, Adam
	momentum (for SGD and RMSprop)	0.1, 0.3, 0.5, 0.7, 0.9
	weight	0, 1e-4, 1e-3
	α (for RMSprop)	0.9, 0.99
	amsgrad (for Adam)	True, False
	dropout ratio	0, 0.1, 0.2, 0.3, 0.4, 0.5
	max_epochs	20, 40, 60, …, 280, 300
	batch_size	32, 64, 128, 256, 516
	criterion	CrossEntropyLoss, NLLLoss

S3. Sensitivity to Iteration Parameters

Table S2. Baseline iteration parameters for sensitivity test

iteration parameter	baseline value	test value
max. number of iterations for selected CI calculation	3	infinity (i.e., fully converged calculation)
max. sampling number of queries	2000	500, 1000, 5000
max. level of excitations	only single excitations	single and double excitations
query sampling method	based on ML prediction probability	without using ML prediction probability
CI coefficient threshold for important configurations	0.01	0.005
ML algorithm	kernel ridge classifier	
scoring method for hyperparameter tuning	F1	
cross-validation (CV)	5-fold for ANN and 10-fold for others	no CV

We have tested two protocol iteration parameters first to accelerate the SCI calculation, which usually is the most time-consuming task in the protocol.

S3.1. Maximum number of iterations for each selected CI calculation

The maximum number of iterations for an SCI calculation can be limited to a small value (i.e., 3) even if the SCI calculation is not fully converged. In this case, only the last SCI calculation in the termination step is performed until fully converged (i.e., setting the "itermax" option in the GAMESS(US) as 1000). Although the average numbers of important configurations identified for naphthalene, anthracene, and tetracene is smaller when using not-converged SCI calculations compared to using fully converged ones (4.40%, 5.85% and 9.24% reductions for naphthalene, anthracene, and tetracene, respectively), the computed excitation energies do not vary significantly. The benefit of using unconverged SCI calculations is that the computation time for the ALCI protocol can be significantly reduced, in particularly as the acene size increases: 68.6%, 34.7% and 5.9% wall time consumed for naphthalene, anthracene and tetracene, respectively (i.e., elapsed time for running ALCI protocol except for the geometry optimization, HF and RASCI (n=2) calculations) compared to using fully converged SCI calculations.

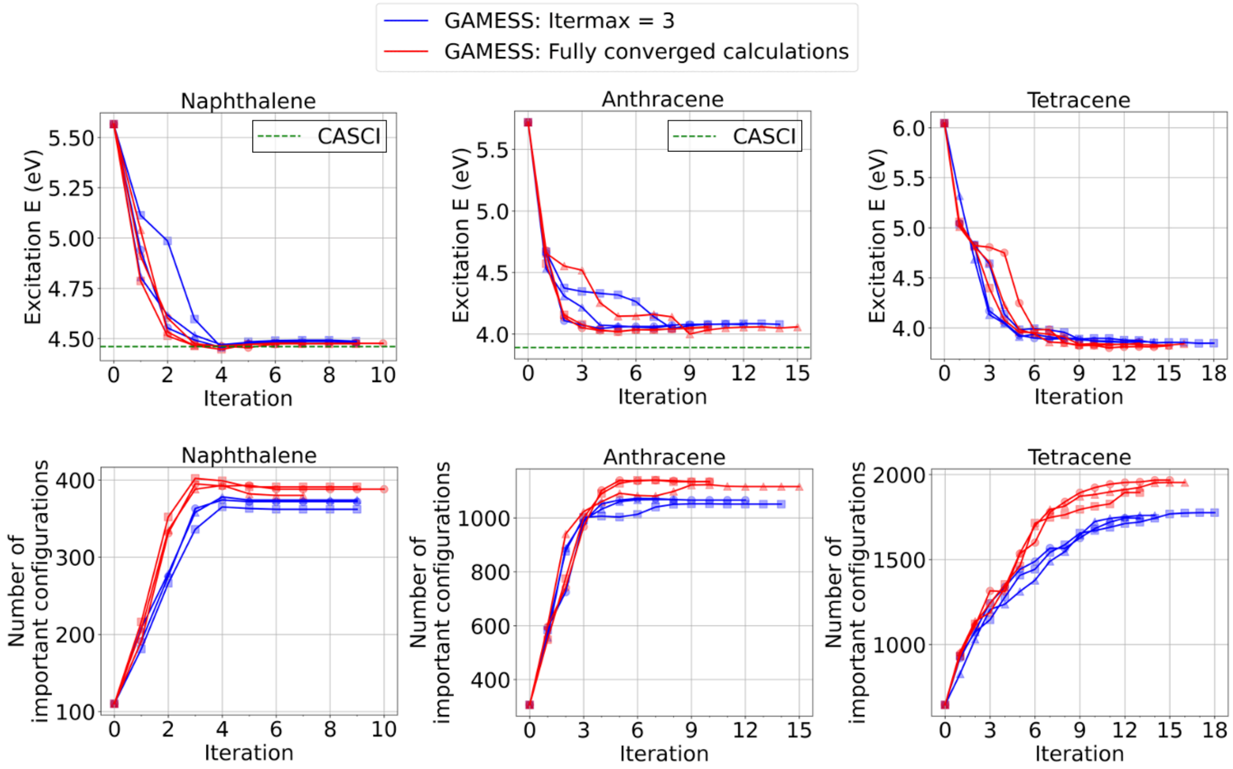

Figure S4. ALCI protocol convergences in terms of excitation energy and the number of important configurations depending on different SCI calculation options for naphthalene, anthracene, and tetracene. Three independent protocol calculations (as indicated with different marker types) were performed. Iteration zero corresponds to the RASCI (n=2) calculation.

Table S3. Iteration results for different SCI calculation options. Average values are in bold.

SCI cal. option	itermax = 3					fully converged SCI calculations				
acene	run	# of iterations	# of important configurations	excitation E (eV)	wall time (s)[a]	run	# of iterations	# of important configurations	excitation E (eV)	wall time (s)[a]
naphthalene (10e, 10o)	1[b]	9	372	4.48	221	1[c]	10	388	4.48	372
	2[b]	9	362	4.49	220	2[c]	9	391	4.47	351
	3[b]	9	374	4.48	224	3[c]	7	380	4.48	270
	**avg.**	**9.0**	**369**	**4.48**	**222**	**avg.**	**8.7**	**386**	**4.48**	**331**
anthracene (14e, 14o)	1[b]	12	1066	4.08	2674	1[c]	10	1135	4.06	6087
	2[b]	14	1051	4.08	3356	2[c]	10	1134	4.06	5592
	3[b]	8	1068	4.07	2030	3[c]	15	1116	4.06	11530
	**avg.**	**11.3**	**1062**	**4.07**	**2687**	**avg.**	**11.7**	**1128**	**4.06**	**7736**
tetracene (18e, 18o)	1[b]	13	1744	3.86	7287	1[c]	15	1967	3.82	181118
	2[b]	18	1775	3.85	12103	2[c]	13	1894	3.85	137138
	3[b]	14	1759	3.86	9490	3[c]	16	1953	3.84	171728
	**avg.**	**15.0**	**1759**	**3.86**	**9627**	**avg.**	**14.7**	**1938**	**3.84**	**163328**

[a]Wall timings for both the iteration and termination steps of the ALCI protocol (i.e., do not include the initialization steps for DFT optimization, HF calculation, and RASCI (n=2)) are provided.
[b]Computed using Intel i7-9700F CPU core (3.00 GHz).
[c]Computed using Intel i9-10980XE CPU core (3.00 GHz).

*SCI calculations and KRC model training/predictions were performed on single CPU core due to the limitation of the software used in this work (i.g, scikit-learn package and GENCI program in the GAMESS package, respectively).

S3.2. Maximum sampling number of queries

To reduce the time for the SCI calculations, one can limit the maximum sampling number of queries from the ML-predicted important configurations. Depending on how many important configurations are ML-predicted at each iteration, the number of configurations used for SCI calculations can vary considerably. If large numbers of configurations are used to in the SCI calculations, then the resulting computational cost can be high and the overall ALCI calculations take a long time. To test whether limiting the number of queries can accelerate the protocol calculations without sacrificing the accuracy, 500, 1000, 2000, and 5000 sampling numbers are tested for anthracene and tetracene (naphthalene was excluded since its query sampling number is smaller than 500). For anthracene, the computed excitation energies are nearly the same (i.e., 4.07–4.13 eV) regardless of the sampling size, and there is no significant saving of computation time (i.e., average 1 hr 16 min. for the 500-query sampling vs. 1h 34 min. for the 5000-query sampling) while the iteration numbers are different (i.e., 27 iterations for the 500-query sampling vs. 10 iterations for the 5000-query sampling). On the other hand, for tetracene, using 5000 queries resulted in a large increase in computation time compared to sampling a smaller number of queries (i.e., average 10 hr vs. 2–3 hr). Note that using too small sampling numbers such as 500 and 1000 queries for tetracene leads to a slightly larger excitation energy because some of the protocol calculations were terminated too early (3.93 and 4.11 eV compared to 3.86–3.87 eV). This may occur if not enough important configurations are added, and the excitation energy is not properly updated. In these cases the protocol misjudges that the excitation energy is converged. Considering a tradeoff between computation time and excitation energy convergence, a 2000-query sampling is used for the default setting.

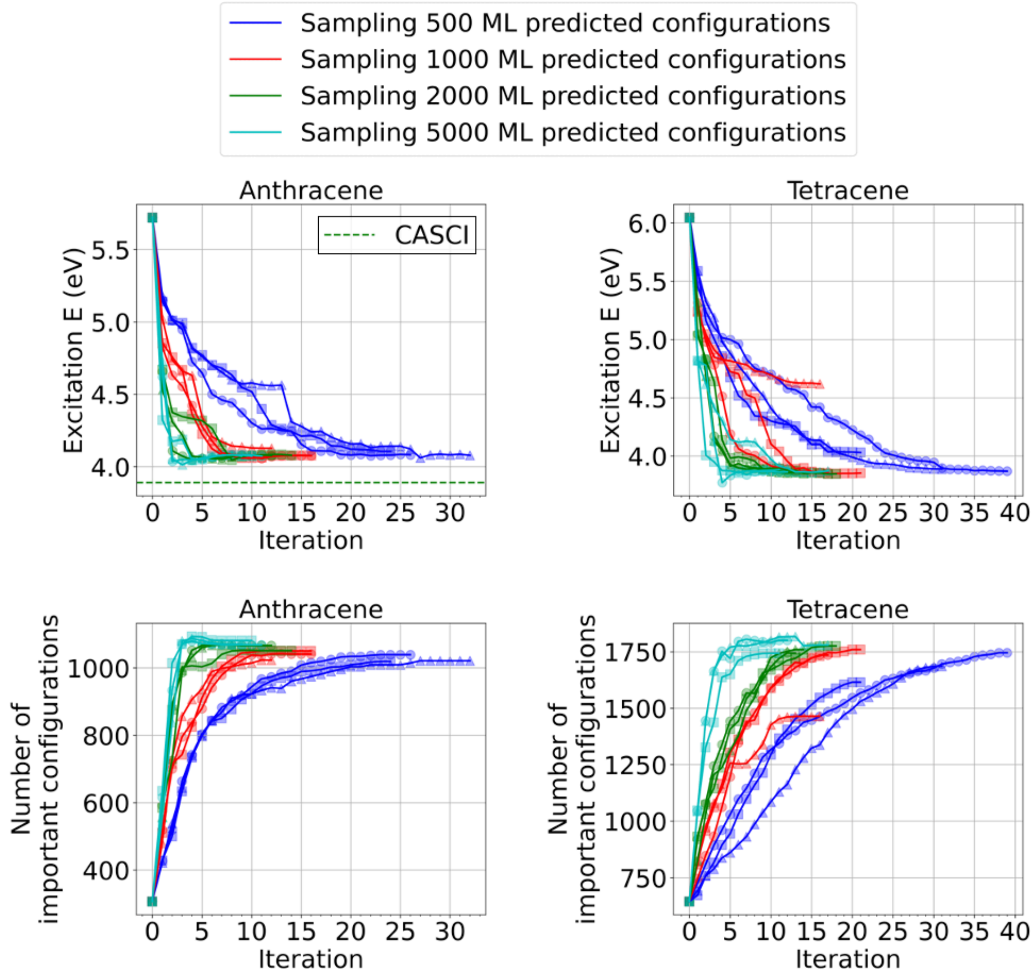

Figure S5. ALCI protocol convergences in terms of excitation energy and the number of important configurations depending on different query sampling numbers for anthracene, and tetracene. Three independent protocol calculations (as indicated with different marker types) were performed. The iteration zero corresponds to the RASCI (n=2) calculation.

Table S4. Iteration results for different maximum sampling number of queries. Average values in bold.

acene	max. sampling # of queries	run	# of iteration	# of important configurations	# of important SDs	excitation E (eV)	wall time (s)	wall time (hh:mm:ss)[a]
anthracene (14e, 14o)	500	1[b]	26	1039	37064	4.08	4324	01:12:04
		2[b]	24	1019	36241	4.11	4246	01:10:46
		3[b]	32	1021	36133	4.08	5175	01:26:15
		**avg.**	**27.3**	**1026**	**36479**	**4.09**	**4582**	**01:16:22**
	1000	1[b]	16	1041	36864	4.08	2445	00:40:45
		2[b]	16	1050	36306	4.08	3047	00:50:47
		3[b]	12	1023	36925	4.13	3035	00:50:35
		**avg.**	**14.7**	**1038**	**36698**	**4.09**	**2842**	**00:47:22**

acene	max. sampling # of queries	run	# of iteration	# of important configurations	# of important SDs	excitation E (eV)	wall time (s)	wall time (hh:mm:ss)[a]
	2000	1[b]	12	1066	38360	4.08	2674	00:44:34
		2[b]	14	1051	37592	4.08	3356	00:55:56
		3[b]	8	1068	37962	4.07	2030	00:33:50
		**avg.**	**11.3**	**1062**	**37971**	**4.07**	**2687**	**00:44:47**
	5000	1[b]	11	1068	38422	4.07	6316	01:45:16
		2[b]	10	1081	38702	4.07	5568	01:32:48
		3[b]	9	1070	38366	4.07	5084	01:24:44
		**avg.**	**10.0**	**1073**	**38497**	**4.07**	**5656**	**01:34:16**
tetracene (18e, 18o)	500	1[b]	39	1745	55445	3.87	14809	04:06:49
		2[b]	21	1615	47318	4.03	6276	01:44:36
		3[b]	31	1685	49249	3.89	8468	02:21:08
		**avg.**	**30.3**	**1682**	**50671**	**3.93**	**9851**	**02:44:11**
	1000	1[b]	17	1735	52027	3.86	10475	02:54:35
		2[b]	21	1759	54237	3.85	13586	03:46:26
		3[b]	16	1464	45299	4.62	8665	02:24:25
		**avg.**	**17.7**	**1653**	**50521**	**4.11**	**10909**	**03:01:49**
	2000	1[b]	13	1744	51694	3.86	7287	02:01:27
		2[b]	18	1775	54391	3.85	12103	03:21:43
		3[b]	14	1759	53554	3.86	9490	02:38:10
		**avg.**	**15.0**	**1759**	**53213**	**3.86**	**9627**	**02:40:27**
	5000	1[b]	12	1800	55152	3.86	29158	08:05:58
		2[b]	11	1743	50822	3.88	20816	05:46:56
		3[c]	17	1779	54084	3.87	58625	16:17:05
		**avg.**	**13.3**	**1774**	**53353**	**3.87**	**36200**	**10:03:20**

[a]Wall timings for both the iteration and termination steps of the ALCI protocol (i.e., do not include the initialization steps for DFT optimization, HF calculation, and RASCI (n=2)) are provided.
[b]Computed using Intel i7-9700F CPU core (3.00 GHz).
[c]Computed using Intel i9-10980XE CPU core (3.00 GHz).
[*]SCI calculations and KRC model training/predictions were performed on single CPU core due to the limitation of the software used in this work (i.g, scikit-learn package and GENCI program in the GAMESS package, respectively).

S3.3. Cross-validation

To avoid overfitting, we adopted cross-validation (CV) to find optimal hyperparameters for the ML model at every iteration, e.g., 5-fold and 10-fold CV for ANN and other ML algorithms, respectively. Using CV most likely increases the training time due to multiple training of the ML models. However, it is not obvious how using or not using CV affects the overall computation time. As a test, we conducted ALCI calculations using KRC, ANN, and XGBoost models with and without CV for naphthalene, tetracene, and hexacene (Table S5). For the smallest system, naphthalene, the ALCI calculations without CV are accelerated compared to those with CV, while both cases converged to the similar number of important configurations and excitation energies. In particular, a huge acceleration is observed when using ANN (i.e., about 15 min. vs. 1 hour and 30 min. without CV and with CV, respectively). On the other hand, when using KRC and XGBoost, the reduction in the overall ALCI cost without CV for naphthalene is only about 18%. The reason is that, as shown in Table S15, the portion of ML training in the ALCI protocol when using CV is much smaller for KRC (i.e., 22%) and XGBoost (25%) compared to ANN (96%). For tetracene, all calculations lead to similar results in terms of number of important configurations and excitation energy, but only ANN exhibits a significant decrease (i.e., 62%) in the overall ALCI computation time because the portion of the ML training is decreased as the system size increases. As shown in Table S15, the ML training cost when using CV for KRC and XGBoost is 11% and 4%, respectively but for ANN it is about 88%. In the case of hexacene, the computed excitation energies are similar, but, unlike for smaller systems, there is no significant reduction in the ALCI cost. Considering the small portion of the ML training part when using CV for KRC (3%) and XGBoost (1%), slight variations of the overall cost for KRC (+13%) and XGBoost (-15%) could be due to the stochastic nature of ML training/predictions. Unexpectedly, there is a small reduction in cost (4%) for ANN when not using CV although about 49% of the overall ALCI cost is due to the training ANN models. A detailed analysis revealed that the decrease in the ML training cost is offset by a non-negligible increase in the last fully converged SCI calculation. The number of important configurations identified via the ALCI protocol increases from 2366 to 2512 when not using CV, possibly due to a poor generalization capability. Although not using CV results in a slight increase in the number of

important configurations (6.1%), the computational cost of the last SCI calculation increases from about 4 hours to 8–9 hours.

Table S5. Iteration results for different cross-validation options. Average values are in bold.

cross-validation	5-fold for ANN, and 10-fold for others					no cross-validation (train:test = 80:20)					Time % (no CV / with CV)
ML algorithm	run	# of iterations	# of important config.	excitation E (eV)	wall time (hh:mm:ss)	run	# of iterations	# of important config.	excitation E (eV)	wall time (hh:mm:ss)	
naphthalene (10e, 10o)											
KRC	1[b]	9	372	4.48	00:03:41	1[c]	8	374	4.48	00:02:29	
	2[b]	9	362	4.49	00:03:40	2[c]	7	376	4.48	00:02:21	
	3[b]	9	374	4.48	00:03:44	3[c]	10	366	4.49	00:03:08	
	**avg.**	**9.0**	**369**	**4.48**	**00:03:42**	**avg.**	**8.3**	**372**	**4.48**	**00:02:39**	**71.6%**
ANN	1[b,d]	13	359	4.50	01:32:11	1[c,e]	8	364	4.50	00:14:34	
	2[b,d]	12	354	4.51	01:29:03	2[c,e]	9	339	4.51	00:15:24	
	3[b,d]	15	354	4.50	01:36:46	3[c,e]	9	367	4.49	00:13:39	
	**avg.**	**13.3**	**356**	**4.50**	**01:32:40**	**avg.**	**8.7**	**357**	**4.50**	**00:14:32**	**15.7%**
XGBoost	1[c]	10	367	4.47	00:03:58	1[c]	11	365	4.47	00:03:21	
	2[c]	9	357	4.49	00:03:32	2[c]	10	368	4.48	00:03:15	
	3[c]	13	361	4.49	00:05:04	3[c]	8	372	4.47	00:02:31	
	**avg.**	**10.7**	**362**	**4.48**	**00:04:11**	**avg.**	**9.7**	**368**	**4.48**	**00:02:59**	**71.3%**
tetracene (18e, 18o)											
KRC	1[b]	13	1744	3.86	02:01:27	1[c]	18	1779	3.86	03:45:43	
	2[b]	18	1775	3.85	03:21:43	2[c]	14	1770	3.87	02:57:25	
	3[b]	14	1759	3.86	02:38:10	3[c]	12	1755	3.87	02:01:41	
	**avg.**	**15.0**	**1759**	**3.86**	**02:40:27**	**avg.**	**14.7**	**1768**	**3.87**	**02:54:56**	**109.0%**
ANN	1[b,d]	11	1396	3.93	04:38:03	1[c,e]	12	1377	3.92	01:29:30	
	2[c,e]	9	1436	3.91	04:41:30	2[c,e]	11	1407	3.93	02:20:56	
	3[b,d]	11	1642	3.88	05:17:19	3[c,e]	12	1420	3.93	01:46:37	
	**avg.**	**10.3**	**1491**	**3.91**	**04:52:17**	**avg.**	**11.7**	**1401**	**3.93**	**01:52:21**	**38.4%**
XGBoost	1[c]	15	1747	3.87	02:36:08	1[c]	15	1763	3.87	03:06:34	
	2[c]	12	1719	3.92	01:57:39	2[c]	17	1768	3.85	03:17:00	
	3[c]	18	1756	3.86	03:09:30	3[c]	17	1750	3.86	02:55:16	
	**avg.**	**15.0**	**1741**	**3.88**	**02:34:26**	**avg.**	**16.3**	**1760**	**3.86**	**03:06:17**	**120.6%**
hexacene (26e, 26o)											
KRC	1[b]	15	2393	2.88	15:19:35	1[c]	12	2383	2.93	12:49:23	
	2[c]	20	2487	2.90	22:25:52	2[c]	17	2529	2.90	23:07:23	
	3[b]	17	2411	2.89	19:51:51	3[c]	18	2608	2.89	29:13:30	
	**avg.**	**17.3**	**2430**	**2.89**	**19:12:26**	**avg.**	**15.7**	**2507**	**2.91**	**21:43:25**	**113.1%**
ANN	1[b,d]	14	2377	2.89	18:20:20	1[c,e]	11	2364	2.89	11:11:11	
	2[c,e]	10	2360	2.90	19:33:04	2[c,e]	16	2609	2.92	23:36:43	
	3[c,e]	13	2362	2.89	19:45:35	3[c,e]	11	2562	2.93	20:34:50	
	**avg.**	**12.3**	**2366**	**2.89**	**19:13:00**	**avg.**	**12.7**	**2512**	**2.91**	**18:27:35**	**96.1%**
XGBoost	1[c]	23	2668	2.92	48:30:00	1[c]	23	2645	2.93	44:39:25	
	2[c]	26	2653	2.92	43:40:17	2[c]	23	2670	2.92	38:02:49	

cross-validation	5-fold for ANN, and 10-fold for others					no cross-validation (train:test = 80:20)					Time % (no CV / with CV)
ML algorithm	run	# of iterations	# of important config.	excitation E (eV)	wall time (hh:mm:ss)	run	# of iterations	# of important config.	excitation E (eV)	wall time (hh:mm:ss)	
naphthalene (10e, 10o)											
	3[c]	23	2688	2.94	57:56:59	3[c]	22	2663	2.95	45:19:39	
	**avg.**	**24.0**	**2670**	**2.93**	**50:02:25**	**avg.**	**23**	**2659**	**2.93**	**42:40:38**	**85.3%**

[a]Wall timings for both the iteration and termination steps of the ALCI protocol (i.e., do not include the initialization steps for DFT optimization, HF calculation, and RASCI (n=2)) are provided. Available for KRC, XGBoost, ANN. To compare computational cost, the number of CPU cores for the calculations were limited to 5 cores if ML model training/predictions can be parallelized (i.e., for XGBoost). KRC models were trained and used with one CPU core due to the limitation of the software.
[b]Computed using Intel i7-9700F CPU core (3.00 GHz).
[c]Computed using Intel i9-10980XE CPU core (3.00 GHz).
[d]Using NVIDIA GeForce RTX 3090 for ANNs.
[e]Using NVIDIA Quadro RTX 8000 for ANNs.
[*]SCI calculations and KRC model training/predictions were performed on single CPU core due to the limitation of the software used in this work (i.g, scikit-learn package and GENCI program in the GAMESS package, respectively).

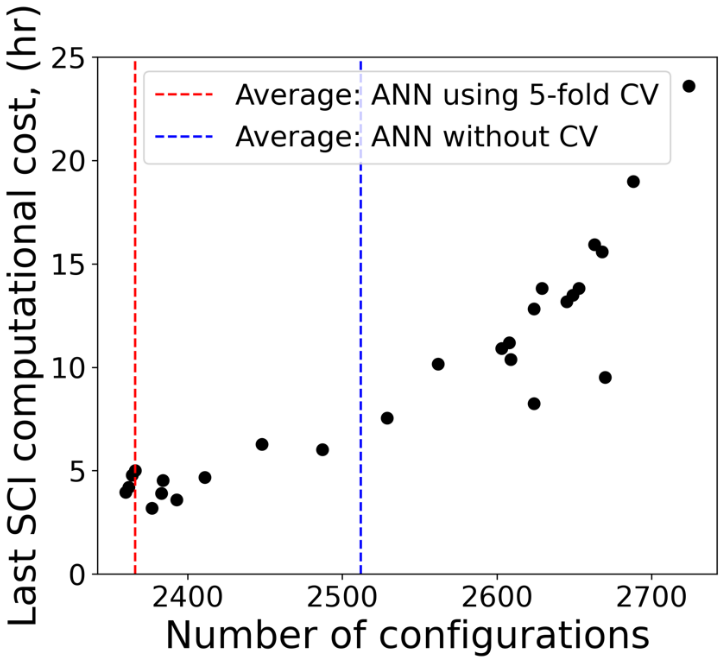

Figure S6. Variations of the computational cost for the last SCI calculation (i.e., fully converged one to obtain accurate excitation energy) depending on the number of configurations for hexacene.

S4. Generation of Excited Configurations

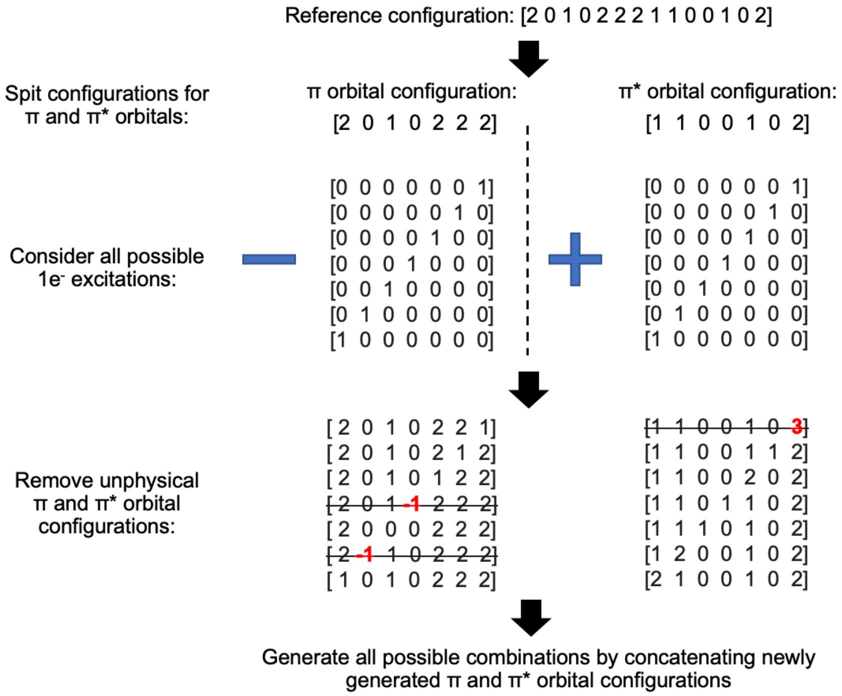

Figure S7. Algorithm for generating singly excited configurations from a given configuration. If multiple configurations are given as reference configurations, all possible excited configurations are generated for each reference configuration, and then only unique configurations are saved. For a higher excitation, multiple single excitations can be executed consecutively.

Table S6. Computational time comparison for generation of excited configurations up to 4th and 5th excitations from the ground state configuration for anthracene and tetracene.

max.excitation level		4		5	
hardware		one CPU[a]	one GPU[b]	one CPU[a]	one GPU[b]
anthracene (14e, 14o)	# of excited configurations	32683		103439	
	time consumed	21 sec.	9 sec.	6 min. 57 sec.	49 sec.
tetracene (18e, 18o)	# of excited configurations	197838		975762	
	time consumed	15 min. 18 sec.	1 min. 23 sec.	11 hr 34 min. 17 sec.	48 min. 53 sec.

[a]Computed using Intel i9-10980XE CPU core (3.00 GHz), NumPy[9] (ver. 1.19.1) Python library was used for handling large data arrays.
[b]Computed using NVIDIA Quadro RTX 8000. CuPy[10] (ver. 8.1.0) Python library was used for handling large data arrays.

S5. Machine Learning Model Performance

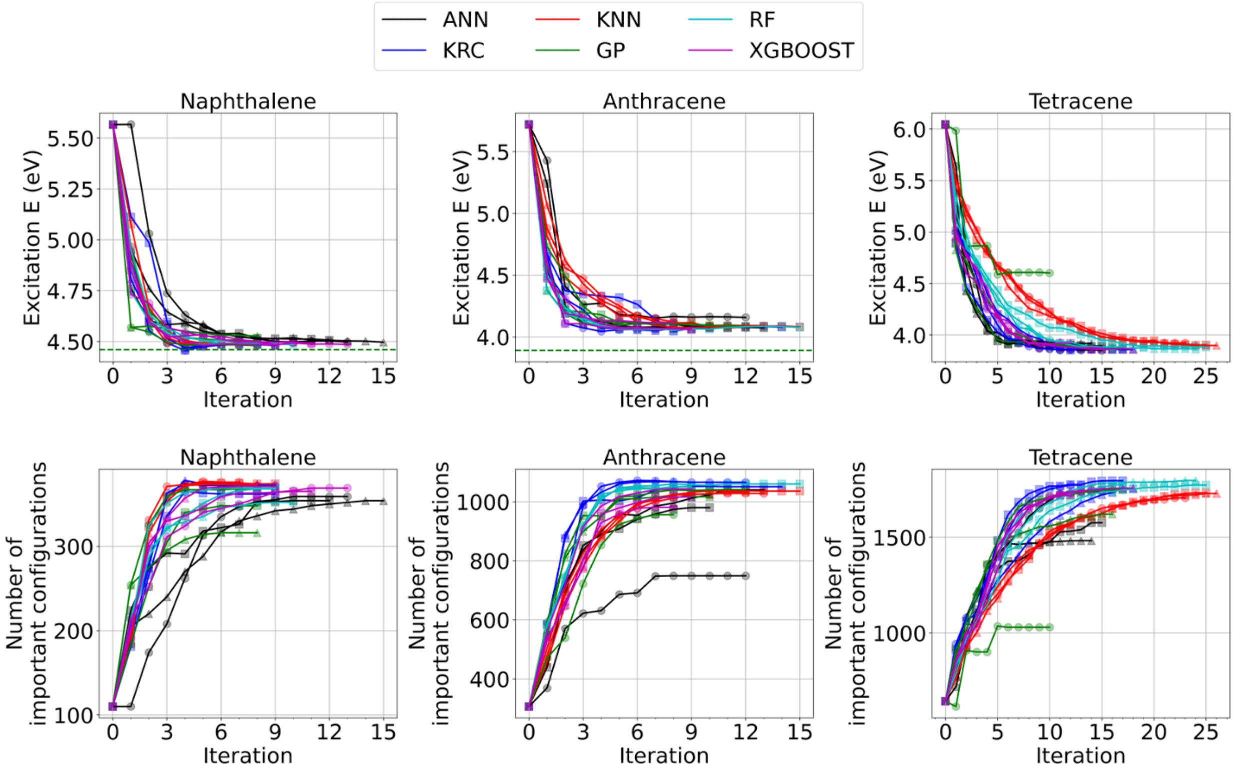

Figure S8. ALCI protocol convergences in terms of excitation energy and the number of important configurations depending on different ML algorithms for naphthalene, anthracene, and tetracene. Three independent protocol calculations (as indicated with different marker types) were performed. Iteration zero corresponds to the RASCI (n=2) calculation. The baseline iteration parameters (except for a type of ML algorithm) listed in Table S2 were used to produce data.

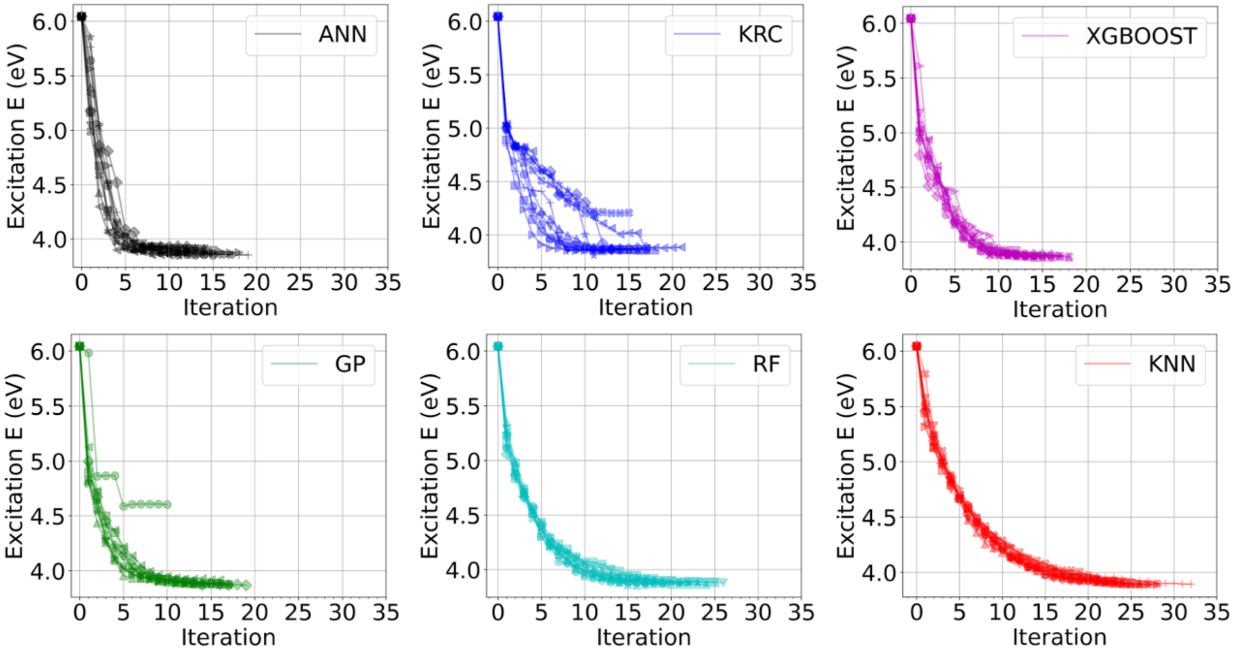

Figure S9. ALCI protocol convergences in terms of excitation energy with different ML algorithms for tetracene. 10 independent calculations (as indicated with different marker types) for each model are performed to show a general trend and reproducibility of the results. Iteration zero corresponds to the RASCI (n=2) calculation. The baseline iteration parameters (except for a type of ML algorithm) listed in Table S2 were used to produce data.

Table S7. Average number of ALCI iterations using different ML algorithms for naphthalene, anthracene, and tetracene.[a] The numbers in the parenthesis are the rankings of each algorithm.

ML algorithm	naphthalene	anthracene	tetracene
KRC	9.0 (3)	11.3 (4)	15.0 (2)
KNN	8.0 (1)	13.3 (6)	26.3 (6)
GP	8.0 (1)	10.0 (2)	15.0 (2)
RF	9.3 (4)	11.0 (3)	16.7 (5)
XGBoost	10.7 (5)	9.7 (1)	15.0 (2)
ANN	13.3 (6)	11.7 (5)	10.3 (1)

[a]Results are average of three independent ALCI calculations.

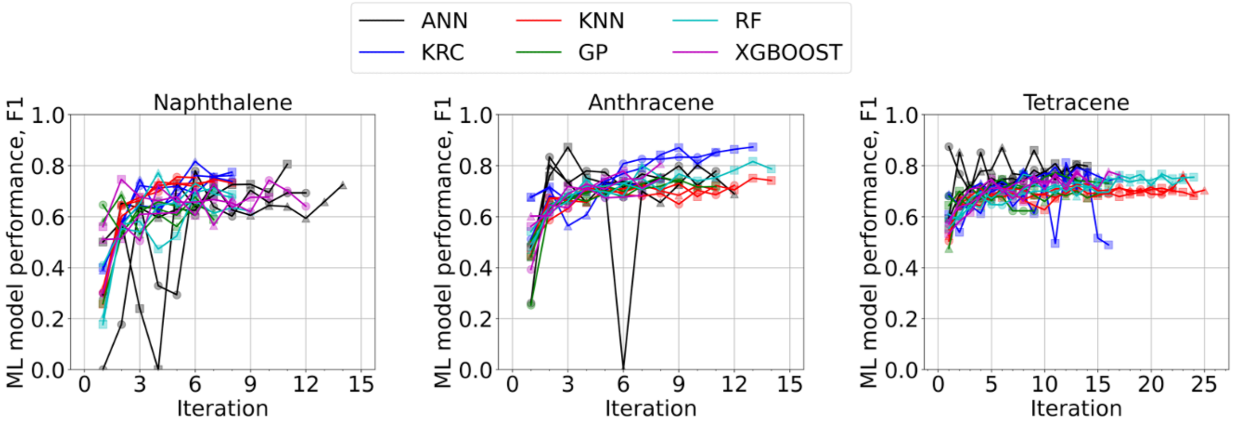

Figure S10. ML model performance (i.e., F1) variations in ALCI calculations for naphthalene, anthracene, and tetracene. The baseline iteration parameters (except for a type of ML algorithm) listed in Table S2 were used to produce data.

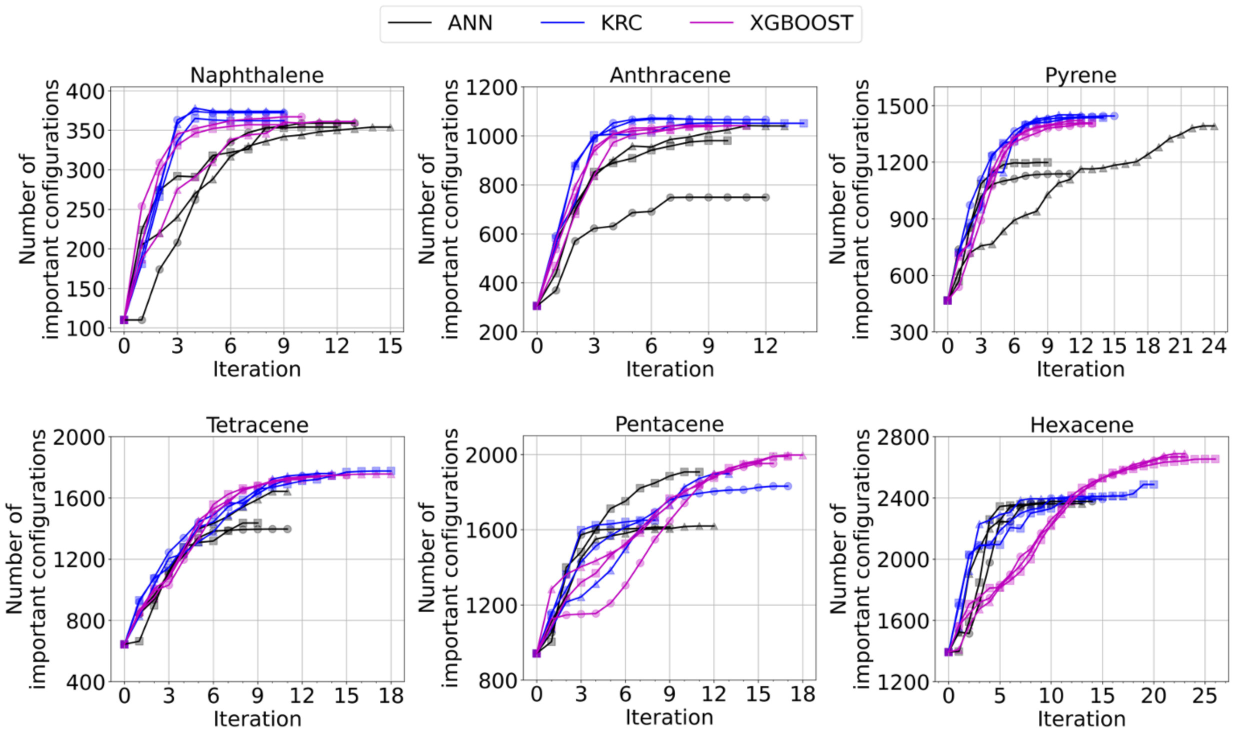

Figure S11. ALCI protocol convergences in terms of the number of important configurations with ANN, KRC, and XGBoost for acenes and pyrene. Three independent calculations (as indicated with different marker types) for each model are performed to show a general trend and reproducibility of the results. The iteration zero corresponds to the RASCI (n=2) calculation. The baseline iteration parameters (except for ML algorithm) listed in Table S2 were used to produce data.

S6. Active Learning CI Protocol Results

Table S8. Default iteration parameters for data production

iteration parameter	value
max. number of iterations for selected CI calculation	3
max. sampling number of queries	2000 (For pentacene and hexacene, 3000 and 5000 are also used to confirm 2000 sampling is sufficient for obtaining converged excitation energy)
max. level of excitations	only single excitations
query sampling method	based on ML prediction probability
CI coefficient threshold for important configurations	0.01
scoring method for hyperparameter tuning	F1

S6.1. Naphthalene

- Excitation energy (CASCI (10e, 10o)) = 4.46 eV (wall time = 20s)
- Total number of configurations in CAS = 8,953
- Total number of configuration state functions (CSFs) in CAS = 19,404
- Total number of determinants in CAS = 63,504

Table S9. ALCI protocol results for naphthalene. Average values in bold.

ML algorithm	threshold for CI coefficient	run	# of iterations	excitation energy (eV)	# of important configurations	# of important SDs	wall time (hh:mm:ss)[a]
KRC	0.01	1[b]	9	4.48	372	4,113	00:03:41
		2[b]	9	4.49	362	4,057	00:03:40
		3[b]	9	4.48	374	4,143	00:03:44
		**avg.**	**9.0**	**4.48**	**369**	**4,104**	**00:03:42**
	0.005	1[b]	9	4.45	726	8,384	00:06:24
		2[b]	8	4.45	714	8,306	00:05:54
		3[b]	7	4.45	726	8,447	00:05:20
		**avg.**	**8.0**	**4.45**	**722**	**8,379**	**00:05:53**
ANN	0.01	1[b,d]	13	4.50	359	3,965	01:32:11
		2[b,d]	12	4.51	354	3,713	01:29:03
		3[b,d]	15	4.50	354	3,805	01:36:46
		**avg.**	**13.3**	**4.50**	**356**	**3,828**	**01:32:40**
	0.005	1[b,d]	8	4.46	694	7,774	00:35:39
		2[b,d]	12	4.46	691	7,856	01:02:52
		3[b,d]	11	4.46	710	8,196	02:43:29
		**avg.**	**10.3**	**4.46**	**698**	**7,942**	**01:27:20**
KNN	0.01	1	7	4.49	376	4,177	-

ML algorithm	threshold for CI coefficient	run	# of iterations	excitation energy (eV)	# of important configurations	# of important SDs	wall time (hh:mm:ss)[a]
		2	9	4.49	374	4,143	-
		3	8	4.49	373	4,145	-
		**avg.**	**8.0**	**4.49**	**374**	**4,155**	**-**
	0.005	1	9	4.45	704	8,058	-
		2	11	4.46	683	7,878	-
		3	8	4.45	706	8,192	-
		**avg.**	**9.3**	**4.45**	**698**	**8,043**	**-**
GP	0.01	1	8	4.48	368	3,993	-
		2	8	4.50	348	3,503	-
		3	8	4.53	316	2,727	-
		**avg.**	**8.0**	**4.50**	**344**	**3,408**	**-**
	0.005	1	8	4.45	719	7,952	-
		2	9	4.48	601	5,936	-
		3	6	4.52	511	4,235	-
		**avg.**	**7.7**	**4.48**	**610**	**6,041**	**-**
RF	0.01	1	9	4.49	369	4,107	-
		2	9	4.49	368	4,097	-
		3	10	4.48	352	3,994	-
		**avg.**	**9.3**	**4.49**	**363**	**4,066**	**-**
	0.005	1	8	4.46	690	7,974	-
		2	10	4.45	709	8,214	-
		3	8	4.47	684	7,870	-
		**avg.**	**8.7**	**4.46**	**694**	**8,019**	**-**
XGBoost	0.01	1[c]	10	4.47	367	4,088	00:03:58
		2[c]	9	4.49	357	4,076	00:03:32
		3[c]	13	4.49	361	4,053	00:05:04
		**avg.**	**10.7**	**4.48**	**362**	**4,072**	**00:04:11**
	0.005	1[c]	11	4.47	655	7,854	00:04:34
		2[c]	8	4.46	659	7,457	00:03:29
		3[c]	10	4.47	673	7,791	00:04:21
		**avg.**	**9.7**	**4.47**	**662**	**7,701**	**00:04:08**

[a]Wall timings for both the iteration and termination steps of the ALCI protocol (i.e., do not include the initialization steps for DFT optimization, HF calculation, and RASCI (n=2)) are provided. Available for KRC, XGBoost and ANN. To compare computational cost, the number of CPU cores for the calculations were limited to 5 cores if ML model training/predictions can be parallelized (i.e., for KNN, RF and XGBoost). GP and KRC models were trained and used with one CPU core due to the limitation of the software. SCI calculations were performed on single CPU core due to the limitation of the GENCI program in the GAMESS package.
[b]Computed using Intel i7-9700F CPU core (3.00 GHz).
[c]Computed using Intel i9-10980XE CPU core (3.00 GHz).
[d]Using NVIDIA GeForce RTX 3090 for ANNs.

S6.2. Anthracene

- Excitation energy (CASCI (14e, 14o)) = 3.89 eV (wall time = 3hr 6min 40s)
- Total number of configurations in CAS = 616,227
- Total number of configuration state functions (CSFs) in CAS = 2,760,615
- Total number of determinants in CAS = 11,778,624

Table S10. ALCI protocol results for anthracene. Average values in bold.

ML algorithm	threshold for CI coefficient	run	# of iterations	excitation energy (eV)	# of important configurations	# of important SDs	wall time (hh:mm:ss)[a]
KRC	0.01	1[b]	12	4.08	1,066	38,360	00:44:34
		2[b]	14	4.08	1,051	37,592	00:55:56
		3[b]	8	4.07	1,068	37,962	00:33:50
		**avg.**	**11.3**	**4.07**	**1,062**	**37,971**	**00:44:47**
	0.005	1[b]	10	3.97	2,493	100,632	01:19:06
		2[b]	14	3.97	2,472	99,781	01:54:05
		3[b]	11	3.98	2,457	100,571	01:47:07
		**avg.**	**11.7**	**3.97**	**2,474**	**100,328**	**01:40:06**
ANN	0.01	1[b,d]	12	4.16	749	9,978	02:15:20
		2[c,e]	10	4.07	980	25,788	03:46:42
		3[c,e]	13	4.07	1,040	34,620	03:50:31
		**avg.**	**11.7**	**4.10**	**923**	**23,462**	**03:17:31**
	0.005	1[b,d]	12	3.97	2,419	86,589	07:25:02
		2[b,d]	9	3.98	2,273	64,920	04:23:18
		3[b,d]	10	3.98	2,366	84,223	04:49:07
		**avg.**	**10.3**	**3.98**	**2,353**	**78,577**	**05:32:29**
KNN	0.01	1	13	4.09	1,028	35,668	-
		2	15	4.08	1,036	36,208	-
		3	12	4.08	1,033	35,621	-
		**avg.**	**13.3**	**4.09**	**1,032**	**35,832**	**-**
	0.005	1	21	3.98	2,404	94,995	-
		2	16	4.00	2,396	94,238	-
		3	16	4.00	2,391	95,616	-
		**avg.**	**17.7**	**4.00**	**2,397**	**94,950**	**-**
GP	0.01	1	8	4.10	956	27,104	-
		2	12	4.08	1,041	36,609	-
		3	10	4.10	1,017	33,903	-
		**avg.**	**10.0**	**4.09**	**1,005**	**32,539**	**-**
	0.005	1	11	4.12	2,256	91,093	-
		2	10	4.04	2,157	84,964	-
		3	8	4.10	1,914	58,158	-
		**avg.**	**9.7**	**4.09**	**2,109**	**78,072**	**-**
RF	0.01	1	9	4.06	1,051	37,735	-
		2	15	4.08	1,060	37,460	-
		3	9	4.06	1,044	37,317	-

ML algorithm	threshold for CI coefficient	run	# of iterations	excitation energy (eV)	# of important configurations	# of important SDs	wall time (hh:mm:ss)[a]
		**avg.**	**11.0**	**4.07**	**1,052**	**37,504**	**-**
	0.005	1	15	3.98	2,438	98,228	-
		2	11	3.99	2,429	98,633	-
		3	18	3.99	2,441	98,421	-
		**avg.**	**14.7**	**3.99**	**2,436**	**98,427**	**-**
XGBoost	0.01	1[c]	9	4.08	1,043	37,021	00:20:45
		2[c]	11	4.06	1,041	37,539	00:23:31
		3[c]	9	4.07	1,040	37,274	00:22:46
		**avg.**	**9.7**	**4.07**	**1,041**	**37,278**	**00:22:21**
	0.005	1[c]	11	4.01	2,271	95,960	00:49:42
		2[c]	12	4.02	2,338	98,606	00:59:01
		3[c]	14	4.00	2,375	98,042	01:10:38
		**avg.**	**12.3**	**4.01**	**2,328**	**97,536**	**00:59:47**

[a]Wall timings for both the iteration and termination steps of the ALCI protocol (i.e., do not include the initialization steps for DFT optimization, HF calculation, and RASCI (n=2)) are provided. Available for KRC, XGBoost, ANN. To compare computational cost, the number of CPU cores for the calculations were limited to 5 cores if ML model training/predictions can be parallelized (i.e., for KNN, RF and XGBoost). GP and KRC models were trained and used with one CPU core due to the limitation of the software. SCI calculations were performed on single CPU core due to the limitation of the GENCI program in the GAMESS package.
[b]Computed using Intel i7-9700F CPU core (3.00 GHz).
[c]Computed using Intel i9-10980XE CPU core (3.00 GHz).
[d]Using NVIDIA GeForce RTX 3090 for ANNs.
[e]Using NVIDIA Quadro RTX 8000 for ANNs.

S6.3. Pyrene

- Excitation energy (CASCI (16e, 16o)) = 3.79 eV (wall time = 91hr 27min 9s)
- Total number of configurations in CAS = 5,196,627
- Total number of configuration state functions (CSFs) in CAS = 34,763,300
- Total number of determinants in CAS = 165,636,900

Table S11. ALCI protocol results for pyrene. Average values in bold.

ML algorithm	threshold for CI coefficient	run	# of iterations	excitation energy (eV)	# of important configurations	# of important SDs	wall time (hh:mm:ss)[a]
KRC	0.01	1[b]	15	4.12	1,445	41,345	01:41:53
		2[b]	14	4.13	1,436	41,325	01:39:07
		3[b]	12	4.13	1,451	43,213	01:20:59
		**avg.**	**13.7**	**4.13**	**1,444**	**41,961**	**01:34:00**
	0.005	1[b]	19	3.98	3,700	164,552	05:51:22
		2[c]	14	3.98	3,615	266,842	05:32:34
		3[c]	17	3.97	3,666	243,724	06:51:00
		**avg.**	**16.7**	**3.98**	**3,660**	**225,039**	**06:04:59**
ANN	0.01	1[c,e]	11	4.16	1,138	23,217	05:33:27
		2[b,d]	9	4.15	1,198	24,096	03:45:22

ML algorithm	threshold for CI coefficient	run	# of iterations	excitation energy (eV)	# of important configurations	# of important SDs	wall time (hh:mm:ss)[a]
		3[b,d]	24	4.13	1,392	89,059	08:21:14
		**avg.**	**14.7**	**4.15**	**1,243**	**45,457**	**05:53:21**
	0.005	1[b,d]	19	4.00	3,304	122,972	10:11:17
		2[c,e]	16	3.98	3,524	199,577	18:13:30
		3[b,d]	12	3.99	3,443	131,976	11:34:55
		**avg.**	**15.7**	**3.99**	**3,424**	**151,508**	**13:19:54**
KNN	0.01	1	20	4.15	1,420	40,125	-
		2	19	4.15	1,396	37,719	-
		3	18	4.15	1,399	38,041	-
		**avg.**	**19.0**	**4.15**	**1,405**	**38,628**	**-**
	0.005	1	29	4.03	3,485	148,242	-
		2	28	4.01	3,484	145,038	-
		3	25	4.04	3,484	149,962	-
		**avg.**	**27.3**	**4.03**	**3,484**	**147,747**	**-**
GP	0.01	1	15	4.15	1,434	38,927	-
		2	18	4.17	1,397	38,753	-
		3	20	4.17	1,422	39,067	-
		**avg.**	**17.7**	**4.17**	**1,418**	**38,916**	**-**
	0.005	1	10	4.12	2,656	81,181	-
		2	18	4.05	3,544	152,955	-
		3	21	4.04	3,604	161,948	-
		**avg.**	**16.3**	**4.07**	**3,268**	**132,028**	**-**
RF	0.01	1	18	4.14	1,433	41,817	-
		2	15	4.12	1,434	42,312	-
		3	14	4.14	1,420	41,733	-
		**avg.**	**15.7**	**4.13**	**1,429**	**41,954**	**-**
	0.005	1	20	3.99	3,611	161,474	-
		2	21	4.01	3,596	160,148	-
		3	21	4.01	3,591	161,577	-
		**avg.**	**20.7**	**4.00**	**3,599**	**161,066**	**-**
XGBoost	0.01	1[c]	13	4.13	1,404	40,292	00:45:12
		2[c]	13	4.14	1,408	40,147	00:49:27
		3[c]	12	4.15	1,409	41,127	00:45:04
		**avg.**	**12.7**	**4.14**	**1,407**	**40,522**	**00:46:34**
	0.005	1[c]	21	4.00	3,498	153,243	03:13:05
		2[c]	20	4.00	3,522	159,217	03:44:52
		3[c]	19	4.00	3,494	155,192	03:47:42
		**avg.**	**20.0**	**4.00**	**3,505**	**155,884**	**03:35:13**

[a]Wall timings for both the iteration and termination steps of the ALCI protocol (i.e., do not include the initialization steps for DFT optimization, HF calculation, and RASCI (n=2)) are provided. Available for KRC, XGBoost, ANN. To compare computational cost, the number of CPU cores for the calculations were limited to 5 cores if ML model training/predictions can be parallelized (i.e., for KNN, RF and XGBoost). GP and KRC models were trained and

used with one CPU core due to the limitation of the software. SCI calculations were performed on single CPU core due to the limitation of the GENCI program in the GAMESS package.
[b]Computed using Intel i7-9700F CPU core (3.00 GHz).
[c]Computed using Intel i9-10980XE CPU core (3.00 GHz).
[d]Using NVIDIA GeForce RTX 3090 for ANNs.
[e]Using NVIDIA Quadro RTX 8000 for ANNs.

S6.4. Tetracene

- Excitation energy (CASCI (18e, 18o)) = N/A
- Total number of configurations in CAS = 44,152,809
- Total number of configuration state functions (CSFs) in CAS = 449,141,836
- Total number of determinants in CAS = 2,363,904,400

Table S12. ALCI protocol results for tetracene. Average values in bold.

ML algorithm	threshold for CI coefficient	run	# of iterations	excitation energy (eV)	# of important configurations	# of important SDs	wall time (hh:mm:ss)[a]
KRC	0.01	1[b]	13	3.86	1,744	51,694	02:01:27
		2[b]	18	3.85	1,775	54,391	03:21:43
		3[b]	14	3.86	1,759	53,554	02:38:10
		**avg.**	**15.0**	**3.86**	**1,759**	**53,213**	**02:40:27**
	0.005	1[d]	21	3.74	4,758	247,880	19:12:07
		2[d]	27	3.75	4,857	260,922	38:07:37
		3[b]	20	3.73	4,749	245,157	18:04:15
		**avg.**	**22.7**	**3.74**	**4,788**	**251,320**	**25:08:00**
ANN	0.01	1[b,d]	11	3.93	1,396	19,716	04:38:03
		2[c,e]	9	3.91	1,436	36,582	04:41:30
		3[b,d]	11	3.88	1,642	41,692	05:17:19
		**avg.**	**10.3**	**3.91**	**1,491**	**32,663**	**04:52:17**
	0.005	1[c,e]	21	3.73	3,984	147,894	25:55:19
		2[c,e]	18	3.72	4,022	201,036	34:45:25
		3[c,e]	15	3.74	3,826	190,926	26:02:21
		**avg.**	**18.0**	**3.73**	**3,944**	**179,952**	**28:54:22**
KNN	0.01	1	23	3.94	1,694	49,776	-
		2	32	3.89	1,735	51,414	-
		3	24	3.91	1,731	52,578	-
		**avg.**	**26.3**	**3.91**	**1,720**	**51,256**	**-**
GP	0.01	1	17	3.93	1,709	49,689	-
		2	16	3.87	1,703	48,731	-
		3	12	3.92	1,516	29,532	-
		**avg.**	**15.0**	**3.91**	**1,643**	**42,651**	**-**
RF	0.01	1	18	3.88	1,773	56,614	-
		2	15	3.93	1,758	56,862	-
		3	17	3.89	1,758	55,970	-
		**avg.**	**16.7**	**3.90**	**1,763**	**56,482**	**-**
XGBoost	0.01	1[c]	15	3.87	1,747	52,864	02:36:08

ML algorithm	threshold for CI coefficient	run	# of iterations	excitation energy (eV)	# of important configurations	# of important SDs	wall time (hh:mm:ss)[a]
		2[c]	12	3.92	1,719	52,554	01:57:39
		3[c]	18	3.86	1,756	53,957	03:09:30
		**avg.**	**15.0**	**3.88**	**1,741**	**53,125**	**02:34:26**
	0.005	1[c]	22	3.75	4,672	244,906	16:51:24
		2[c]	23	3.75	4,590	226,451	16:35:12
		3[c]	28	3.75	4,663	232,510	18:47:04
		**avg.**	**24.3**	**3.75**	**4,642**	**234,622**	**17:24:33**

[a]Wall timings for both the iteration and termination steps of the ALCI protocol (i.e., do not include the initialization steps for DFT optimization, HF calculation, and RASCI (n=2)) are provided. Available for KRC, XGBoost, ANN. To compare computational cost, the number of CPU cores for the calculations were limited to 5 cores if ML model training/predictions can be parallelized (i.e., for KNN, RF and XGBoost). GP and KRC models were trained and used with one CPU core due to the limitation of the software. SCI calculations were performed on single CPU core due to the limitation of the GENCI program in the GAMESS package.
[b]Computed using Intel i7-9700F CPU core (3.00 GHz).
[c]Computed using Intel i9-10980XE CPU core (3.00 GHz).
[d]Using NVIDIA GeForce RTX 3090 for ANNs.
[e]Using NVIDIA Quadro RTX 8000 for ANNs.

S6.5. Pentacene

- Excitation energy (CASCI (22e, 22o)) = N/A
- Total number of configurations in CAS = 3,241,135,527
- Total number of configuration state functions (CSFs) in CAS = 79,483,257,308
- Total number of determinants in CAS = 497,634,306,624

Table S13. ALCI protocol results for pentacene. Average values in bold.

ML algorithm	threshold for CI coefficient	query sampling size	run	# of iterations	excitation energy (eV)	# of important configurations	# of important SDs	wall time (hh:mm:ss)[a]
KRC	0.01	2000	1[c]	17	3.44	1,831	33,876	04:58:16
			2[c]	8	3.61	1,650	20,933	02:17:38
			3[b]	13	3.46	1,897	39,664	06:04:25
			**avg.**	**12.7**	**3.50**	**1,793**	**31,491**	**04:26:46**
		3000	1[c]	8	3.47	1,616	16,303	02:01:43
			2[c]	9	3.55	1,576	16,305	01:45:22
			3[b]	13	3.45	1,790	32,659	04:11:20
			**avg.**	**10.0**	**3.49**	**1,661**	**21,756**	**02:39:28**
		5000	1[b]	12	3.49	1,568	14,827	01:40:10
			2[c]	18	3.44	2,056	53,303	32:42:42
			3[c]	14	3.46	2,092	58,953	28:10:12
			**avg.**	**14.7**	**3.46**	**1,905**	**42,361**	**20:51:01**
	0.005	2000	1[c]	27	3.45	5,058	247,707	73:38:44
			2[c]	17	3.47	4,264	165,652	17:12:30
			3[c]	31	3.46	5,018	236,676	62:09:43
			**avg.**	**25.0**	**3.46**	**4,780**	**216,678**	**51:00:19**

ML algorithm	threshold for CI coefficient	query sampling size	run	# of iterations	excitation energy (eV)	# of important configurations	# of important SDs	wall time (hh:mm:ss)[a]
ANN	0.01	2000	1[b,d]	9	3.42	1,613	15,360	04:33:12
			2[c,e]	11	3.45	1,907	36,455	09:15:05
			3[c,e]	12	3.45	1,619	16,051	06:31:03
			**avg.**	**10.7**	**3.44**	**1,713**	**22,622**	**06:46:27**
	0.005		1[c,e]	19	3.48	5,300	284,377	77:56:14
			2[c,e]	11	3.47	2,442	23,079	10:41:09
			3[c,e]	18	3.48	4,843	218,553	49:58:55
			**avg.**	**16.0**	**3.48**	**4,195**	**175,336**	**46:12:06**
XGBoost	0.01	2000	1[c]	16	3.46	1,952	43,718	09:00:26
			2[c]	17	3.47	1,989	49,785	13:35:38
			3[c]	18	3.47	1,997	48,891	11:11:22
			**avg.**	**17.0**	**3.47**	**1,979**	**47,465**	**11:15:49**
	0.005		1[c]	28	3.46	4,964	238,588	72:41:41
			2[c]	25	3.46	5,015	239,157	65:30:26
			3[c]	22	3.46	4,845	217,769	31:41:28
			**avg.**	**25.0**	**3.46**	**4,941**	**231,838**	**56:37:52**

[a]Wall timings for both the iteration and termination steps of the ALCI protocol (i.e., do not include the initialization steps for DFT optimization, HF calculation, and RASCI (n=2)) are provided. To compare computational cost, the number of CPU cores for the calculations were limited to 5 cores if ML model training/predictions can be parallelized (i.e., for XGBoost). KRC models were trained and used with one CPU core due to the limitation of the software. SCI calculations were performed on single CPU core due to the limitation of the GENCI program in the GAMESS package.
[b]Computed using Intel i7-9700F CPU core (3.00 GHz).
[c]Computed using Intel i9-10980XE CPU core (3.00 GHz).
[d]Using NVIDIA GeForce RTX 3090 for ANNs.
[e]Using NVIDIA Quadro RTX 8000 for ANNs.

S6.6. Hexacene

- Excitation energy (CASCI (26e, 26o)) = N/A
- Total number of configurations in CAS = 241,813,226,151
- Total number of configuration state functions (CSFs) in CAS = 14,901,311,070,000
- Total number of determinants in CAS = 108,172,480,360,000

Table S14. ALCI protocol results for hexacene. Average values in bold.

ML algorithm	threshold for CI coefficient	query sampling size	run	# of iterations	excitation energy (eV)	# of important configurations	# of important SDs	wall time (hh:mm:ss)[a]
KRC	0.01	2000	1[b]	15	2.88	2,393	25,369	15:19:35
			2[c]	20	2.90	2,487	31,034	22:25:52
			3[b]	17	2.89	2,411	26,000	19:51:51
			**avg.**	**17.3**	**2.89**	**2,430**	**27,468**	**19:12:26**
		3000	1[c]	13	3.04	2,428	31,350	13:44:43
			2[c]	20	2.94	2,355	24,965	14:49:31
			3[b]	17	2.90	2,629	40,858	30:45:28

ML algorithm	threshold for CI coefficient	query sampling size	run	# of iterations	excitation energy (eV)	# of important configurations	# of important SDs	wall time (hh:mm:ss)[a]
			**avg.**	**16.7**	**2.96**	**2,471**	**32,391**	**19:46:34**
		5000	1[c]	13	3.21	2,598	42,028	58:24:14
			2[c]	8	2.88	2,560	36,442	26:50:18
			3[c]	15	2.90	2,724	53,172	66:42:36
			**avg.**	**12.0**	**3.00**	**2,627**	**43,881**	**41:07:02**
	0.005	2000	1[c]	19	2.99	4,118	57,437	22:02:45
			2[c]	23	3.05	4,228	76,151	50:56:11
			3[c]	14	3.00	3,836	41,122	17:02:41
			**avg.**	**18.7**	**3.02**	**4,061**	**58,237**	**44:54:51**
ANN	0.01	2000	1[b,d]	14	2.89	2,377	24,931	18:20:20
			2[c,e]	10	2.90	2,360	24,715	19:33:04
			3[c,e]	13	2.89	2,362	24,634	19:45:35
			**avg.**	**12.3**	**2.89**	**2,366**	**24,760**	**19:13:00**
	0.005		1[c,e]	9	3.04	3,581	34,487	20:46:52
			2[c,e]	16	3.02	4,688	92,829	42:49:05
			3[c,e]	20	3.03	5,452	175,729	71:08:36
			**avg.**	**15.0**	**3.03**	**4,574**	**101,015**	**44:54:51**
XGBoost	0.01	2000	1[c]	23	2.92	2,668	50,441	48:30:00
			2[c]	26	2.92	2,653	48,662	43:40:17
			3[c]	23	2.94	2,688	54,638	57:56:59
			**avg.**	**24.0**	**2.93**	**2,670**	**51,247**	**50:02:25**
	0.005		1[c]	35	3.04	5,782	224,239	137:04:30
			2[c]	34	3.01	5,977	249,410	186:25:56
			3[c]	40	3.04	6,097	262,954	217:39:18
			**avg.**	**36.3**	**3.03**	**5,952**	**245,534**	**180:10:45**

[a]Wall timings for both the iteration and termination steps of the ALCI protocol (i.e., do not include the initialization steps for DFT optimization, HF calculation, and RASCI (n=2)) are provided. To compare computational cost, the number of CPU cores for the calculations were limited to 5 cores if ML model training/predictions can be parallelized (i.e., for XGBoost). KRC models were trained and used with one CPU core due to the limitation of the software. SCI calculations were performed on single CPU core due to the limitation of the GENCI program in the GAMESS package.
[b]Computed using Intel i7-9700F CPU core (3.00 GHz).
[c]Computed using Intel i9-10980XE CPU core (3.00 GHz).
[d]Using NVIDIA GeForce RTX 3090 for ANNs.
[e]Using NVIDIA Quadro RTX 8000 for ANNs.

S6.7. Computational cost for ML and SCI parts of ALCI protocol calculations

Table S15. Examples of timings for ALCI protocol calculations for different systems

acene	ML algorithm	# of iterations	wall time [hh:mm:ss][a]		
			total	ML model training/predictions	selected CI cal.

naphthalene (10e, 10o)	KRC[b]	9	00:03:40	00:00:48 (21.8%)	00:02:43 (74.1%)
	ANN[b,d]	12	01:29:03	01:25:32 (96.1%)	00:03:17 (3.7%)
	XGBoost[c]	9	00:03:32	00:00:53 (25.0%)	00:02:26 (68.9%)
anthracene (14e, 14o)	KRC[b]	8	00:33:50	00:04:27 (13.2%)	00:29:04 (85.9%)
	ANN[b,d]	12	02:15:20	02:05:31 (92.7%)	00:09:30 (7.0%)
	XGBoost[c]	9	00:20:45	00:02:25 (11.6%)	00:18:06 (87.2%)
tetracene (18e, 18o)	KRC[b]	13	02:01:27	00:12:43 (10.5%)	01:48:07 (89.0%)
	ANN[b,d]	11	04:38:03	04:04:51 (88.1%)	00:32:44 (11.8%)
	XGBoost[c]	12	01:57:39	00:04:27 (3.8%)	01:52:37 (95.7%)
pentacene (22e, 22o)	KRC[b]	13	06:04:25	00:15:43 (4.3%)	05:47:51 (95.5%)
	ANN[b,d]	9	04:33:12	03:27:16 (75.9%)	01:05:23 (23.9%)
	XGBoost[c]	16	09:00:26	00:07:33 (1.4%)	08:51:52 (98.4%)
hexacene (26e, 26o)	KRC[b]	15	15:19:35	00:29:43 (3.2%)	14:48:14 (96.6%)
	ANN[b,d]	14	18:20:20	08:55:33 (48.7%)	09:23:16 (51.2%)
	XGBoost[c]	26	43:40:17	00:21:57 (0.8%)	43:15:14 (99.0%)

[a]Wall timings for both the iteration and termination steps of the ALCI protocol (i.e., do not include the initialization steps for DFT optimization, HF calculation, and RASCI (n=2)) are provided. To compare computational cost, the number of CPU cores for the calculations were limited to 5 cores if ML model training/predictions can be parallelized (i.e., for XGBoost). KRC models were trained and used with one CPU core due to the limitation of the software. SCI calculations were performed on single CPU core due to the limitation of the GENCI program in the GAMESS package.

[b]Computed using Intel i7-9700F CPU core (3.00 GHz).

[c]Computed using Intel i9-10980XE CPU core (3.00 GHz).

[d]Using NVIDIA GeForce RTX 3090 for ANNs.

S7. Comparison of ALCI and CASCI results

S7.1. Comparison of ALCI and CASCI wave functions

Table S16. Active orbitals occupation numbers for ground and excited states obtained with ALCI (using KRC algorithm and 0.01 threshold for coefficient) and CASCI (14e, 14o) for anthracene

	1	2	3	4	5	6	7	8	9	10	11	12	13	14
Ground CASCI	1.977	1.969	1.951	1.947	1.926	1.912	1.867	0.139	0.091	0.074	0.052	0.049	0.029	0.018
Ground ALCI	1.985	1.978	1.965	1.963	1.948	1.934	1.904	0.100	0.068	0.052	0.036	0.035	0.020	0.012
Excited CASCI	1.961	1.952	1.936	1.909	1.892	1.462	1.460	0.568	0.535	0.098	0.094	0.061	0.042	0.028
Excited ALCI	1.975	1.968	1.959	1.940	1.920	1.471	1.467	0.555	0.526	0.071	0.063	0.039	0.027	0.017

Table S17. Active orbitals occupation numbers for ground and excited states obtained with ALCI (using KRC algorithm and 0.01 threshold for coefficient) and CASCI (16e, 16o) for pyrene

	1	2	3	4	5	6	7	8	9	10	11	12	13	14	15	16
Ground CASCI	1.978	1.971	1.962	1.949	1.932	1.930	1.905	1.872	0.134	0.098	0.072	0.067	0.052	0.035	0.027	0.017
Ground ALCI	1.986	1.981	1.975	1.967	1.955	1.954	1.934	1.914	0.091	0.068	0.046	0.045	0.034	0.023	0.017	0.011
Excited CASCI	1.968	1.954	1.945	1.918	1.902	1.900	1.499	1.432	0.558	0.535	0.097	0.096	0.084	0.046	0.041	0.023
Excited ALCI	1.983	1.974	1.967	1.953	1.939	1.937	1.517	1.437	0.559	0.507	0.061	0.058	0.048	0.028	0.022	0.012

S7.2. Comparison of ALCI and CASCI important configurations

Table S18. Number of important configurations and determinants identified via the ALCI protocol compared to the CASCI calculation for naphthalene[a]

acene	ML algorithm	run	number of configurations	number of determinants
CASCI			8,953	63,504
ALCI	KRC	1	372 (4.2%)	4,113 (6.5%)
		2	362 (4.0%)	4,057 (6.4%)
		3	374 (4.2%)	4,143 (6.5%)
		**avg.**	**369.3 (4.1%)**	**4,104 (6.5%)**
	ANN	1	359 (4.0%)	3,965 (6.2%)
		2	354 (4.0%)	3,713 (5.9%)
		3	354 (4.0%)	3,805 (6.0%)
		**avg.**	**355.7 (4.0%)**	**3,828 (6.0%)**
	XGBoost	1	367 (4.1%)	4,088 (6.4%)
		2	357 (4.0%)	4,076 (6.4%)
		3	361 (4.0%)	4,053 (6.4%)
		**avg.**	**361.7 (4.0%)**	**4,072 (6.4%)**

[a]Threshold for CI coefficient = 0.01

To compare how many important configurations are the same in CASCI and ALCI calculations, all CASCI determinants and their CI coefficients should be obtained first. However, CASCI calculations using GAMESS(US) are very time consuming if one uses a tighter printout-tolerance for CI coefficients (i.e., prttol) than using the default value of 0.05. We were able to print all the determinants and their coefficients only for naphthalene. Table S19 shows that how many important configurations and determinants identified via the ALCI protocol can also be found in the top 100, 200, and 300 CASCI important configurations (i.e., have the largest 100, 200, and 300 CI coefficients). It clearly shows that the ALCI calculations can indeed find important configurations (and determinants) up to the top 200 CASCI important configurations. Even for top 300 CASCI configurations, only a few (i.e., 7–13, about 4%) of important configurations is missing when using ALCI calculations.

Table S19. Comparison of top CASCI and ALCI important configurations for naphthalene[a]

acene	ML algorithm	run	number of configurations			number of determinants		
reference CASCI configurations			top 100	top 200	top 300	top 100	top 200	top 300
ALCI	KRC	1	100	200	293 (97.7%)	1,366	2,522	3,473 (97.3%)
		2	100	200	291 (97.0%)	1,366	2,522	3,465 (97.1%)
		3	100	200	295 (98.3%)	1,366	2,522	3,485 (97.6%)
		**avg.**	**100.0**	**200.0**	**293.0 (97.7%)**	1,366	2,522	**3,474 (97.3%)**
	ANN	1	100	200	293 (97.7%)	1,366	2,522	3,395 (95.1%)
		2	99 (99%)	198 (99%)	282 (94.0%)	1,296 (94.9%)	2,382 (94.5%)	3,145 (88.1%)
		3	100	200	287 (95.7%)	1,366	2,522	3,253 (91.1%)
		**avg.**	**99.7 (99.7%)**	**199.3 (99.7%)**	**287.3 (95.8%)**	**1,343 (98.3%)**	**2,475 (98.2%)**	**3,264 (91.4%)**
	XGBoost	1	100	200	291 (97.0%)	1,366	2,522	3,466 (97.1%)
		2	100	200	289 (96.3%)	1,366	2,522	3,458 (96.9%)
		3	100	199 (99.5%)	289 (96.3%)	1,366	2,520 (99.9%)	3,443 (96.4%)
		**avg.**	**100.0**	**199.7 (99.8%)**	**289.7 (96.6%)**	1,366	**2,521 (100%)**	**3,456 (98.8%)**

[a]Threshold for CI coefficient = 0.01. There are 496 important configurations in the CASCI calculation for naphthalene.

S8. Computational methods for the CASCI+PT2 calculations

CASCI+PT2 calculations were performed with the Openmolcas package.[11] The complete active space CI (CASCI) method was employed, followed by second order perturbation theory (CASPT2[12,13]) to include dynamical correlation. All-electron basis sets of atomic natural orbital type with relativistic core corrections (ANO-RCC) were used[14] employing a double-ζ plus polarization basis set (VDZP) for both H and C atoms. The two-electron integral evaluation was simplified using the Cholesky decomposition technique.[15] Scalar relativistic effects were included by means of the Douglas−Kroll−Hess Hamiltonian.[16] The CASPT2 calculations were performed using an IPEA shift of 0.25 a.u. and an imaginary shift of 0.2 a.u. During the CASPT2 calculations, the first 10 and 14 orbitals were frozen for the naphthalene and anthracene molecules, respectively.

References

(1) Gordon, M. S.; Schmidt, M. W. Advances in Electronic Structure Theory: GAMESS a Decade Later. In *Theory and applications of computational chemistry*; Elsevier, 2005; pp 1167–1189.

(2) Schmidt, M. W.; Baldridge, K. K.; Boatz, J. A.; Elbert, S. T.; Gordon, M. S.; Jensen, J. H.; Koseki, S.; Matsunaga, N.; Nguyen, K. A.; Su, S. General Atomic and Molecular Electronic Structure System. *J. Comput. Chem.* **1993**, *14* (11), 1347–1363.

(3) McKinney, W.; others. Data Structures for Statistical Computing in Python. In *Proceedings of the 9th Python in Science Conference*; 2010; Vol. 445, pp 51–56.

(4) Pedregosa, F.; Varoquaux, G.; Gramfort, A.; Michel, V.; Thirion, B.; Grisel, O.; Blondel, M.; Prettenhofer, P.; Weiss, R.; Dubourg, V. Scikit-Learn: Machine Learning in Python. *J. Mach. Learn. Res.* **2011**, *12*, 2825–2830.

(5) Chen, T.; Guestrin, C. XGBoost: A Scalable Tree Boosting System. In *Proceedings of the 22Nd ACM SIGKDD International Conference on Knowledge Discovery and Data Mining*; KDD '16; ACM: New York, NY, USA, 2016; pp 785–794.

(6) Tietz, M.; Fan, T. J.; Nouri, D.; Bossan, B.; skorch Developers. Skorch: A Scikit-Learn Compatible Neural Network Library That Wraps PyTorch. July 2017. https://skorch.readthedocs.io/en/stable/

(7) Paszke, A.; Gross, S.; Massa, F.; Lerer, A.; Bradbury, J.; Chanan, G.; Killeen, T.; Lin, Z.; Gimelshein, N.; Antiga, L.; Desmaison, A.; Kopf, A.; Yang, E.; DeVito, Z.; Raison, M.; Tejani, A.; Chilamkurthy, S.; Steiner, B.; Fang, L.; Bai, J.; Chintala, S. PyTorch: An Imperative Style, High-Performance Deep Learning Library. *Adv. Neural Inf. Process. Syst.* **2019**, 8026–8037.

(8) Bergstra, J.; Komer, B.; Eliasmith, C.; Yamins, D.; Cox, D. D. Hyperopt: A Python Library for Model Selection and Hyperparameter Optimization. *Comput. Sci. Discov.* **2015**, *8*, 14008.

(9) Harris, C. R.; Millman, K. J.; van der Walt, S. J.; Gommers, R.; Virtanen, P.; Cournapeau, D.; Wieser, E.; Taylor, J.; Berg, S.; Smith, N. J.; Kern, R.; Picus, M.; Hoyer, S.; van Kerkwijk, M. H.;

Brett, M.; Haldane, A.; del Río, J. F.; Wiebe, M.; Peterson, P.; Gérard-Marchant, P.; Sheppard, K.; Reddy, T.; Weckesser, W.; Abbasi, H.; Gohlke, C.; Oliphant, T. E. Array Programming with NumPy. *Nature* **2020**, *585*, 357–362.

(10) Okuta, R.; Unno, Y.; Nishino, D.; Hido, S.; Loomis, C. CuPy: A NumPy-Compatible Library for NVIDIA GPU Calculations. In *Proceedings of Workshop on Machine Learning Systems (LearningSys) in The Thirty-first Annual Conference on Neural Information Processing Systems (NIPS)*; 2017.

(1) Gordon, M. S.; Schmidt, M. W. Advances in Electronic Structure Theory: GAMESS a Decade Later. In *Theory and applications of computational chemistry*; Elsevier, 2005; pp 1167–1189.

(2) Schmidt, M. W.; Baldridge, K. K.; Boatz, J. A.; Elbert, S. T.; Gordon, M. S.; Jensen, J. H.; Koseki, S.; Matsunaga, N.; Nguyen, K. A.; Su, S. General Atomic and Molecular Electronic Structure System. *J. Comput. Chem.* **1993**, *14* (11), 1347–1363.

(3) McKinney, W.; others. Data Structures for Statistical Computing in Python. In *Proceedings of the 9th Python in Science Conference*; 2010; Vol. 445, pp 51–56.

(4) Pedregosa, F.; Varoquaux, G.; Gramfort, A.; Michel, V.; Thirion, B.; Grisel, O.; Blondel, M.; Prettenhofer, P.; Weiss, R.; Dubourg, V. Scikit-Learn: Machine Learning in Python. *J. Mach. Learn. Res.* **2011**, *12*, 2825–2830.

(5) Chen, T.; Guestrin, C. XGBoost: A Scalable Tree Boosting System. In *Proceedings of the 22Nd ACM SIGKDD International Conference on Knowledge Discovery and Data Mining*; KDD ’16; ACM: New York, NY, USA, 2016; pp 785–794. https://doi.org/10.1145/2939672.2939785.

(6) Tietz, M.; Fan, T. J.; Nouri, D.; Bossan, B.; skorch Developers. Skorch: A Scikit-Learn Compatible Neural Network Library That Wraps PyTorch. July 2017.

(7) Paszke, A.; Gross, S.; Massa, F.; Lerer, A.; Bradbury, J.; Chanan, G.; Killeen, T.; Lin, Z.; Gimelshein, N.; Antiga, L.; Desmaison, A.; Kopf, A.; Yang, E.; DeVito, Z.; Raison, M.; Tejani, A.; Chilamkurthy, S.; Steiner, B.; Fang, L.; Bai, J.; Chintala, S. PyTorch: An Imperative Style, High-Performance Deep Learning Library. In *Advances in Neural Information Processing Systems*

32; Wallach, H., Larochelle, H., Beygelzimer, A., d\textquotesingle Alché-Buc, F., Fox, E., Garnett, R., Eds.; Curran Associates, Inc., 2019; pp 8024–8035.

(8) Bergstra, J.; Komer, B.; Eliasmith, C.; Yamins, D.; Cox, D. D. Hyperopt: A Python Library for Model Selection and Hyperparameter Optimization. *Comput. Sci. Discov.* **2015**, *8* (1), 14008. https://doi.org/10.1088/1749-4699/8/1/014008.

(9) Harris, C. R.; Millman, K. J.; van der Walt, S. J.; Gommers, R.; Virtanen, P.; Cournapeau, D.; Wieser, E.; Taylor, J.; Berg, S.; Smith, N. J.; Kern, R.; Picus, M.; Hoyer, S.; van Kerkwijk, M. H.; Brett, M.; Haldane, A.; del Río, J. F.; Wiebe, M.; Peterson, P.; Gérard-Marchant, P.; Sheppard, K.; Reddy, T.; Weckesser, W.; Abbasi, H.; Gohlke, C.; Oliphant, T. E. Array Programming with NumPy. *Nature* **2020**, *585* (7825), 357–362. https://doi.org/10.1038/s41586-020-2649-2.

(10) Okuta, R.; Unno, Y.; Nishino, D.; Hido, S.; Loomis, C. CuPy: A NumPy-Compatible Library for NVIDIA GPU Calculations. In *Proceedings of Workshop on Machine Learning Systems (LearningSys) in The Thirty-first Annual Conference on Neural Information Processing Systems (NIPS)*; 2017.

(11) Fdez. Galván, I.; Vacher, M.; Alavi, A.; Angeli, C.; Aquilante, F.; Autschbach, J.; Bao, J. J.; Bokarev, S. I.; Bogdanov, N. A.; Carlson, R. K.; Chibotaru, L. F.; Creutzberg, J.; Dattani, N.; Delcey, M. G.; Dong, S. S.; Dreuw, A.; Freitag, L.; Frutos, L. M.; Gagliardi, L.; Gendron, F.; Giussani, A.; González, L.; Grell, G.; Guo, M.; Hoyer, C. E.; Johansson, M.; Keller, S.; Knecht, S.; Kovačević, G.; Källman, E.; Li Manni, G.; Lundberg, M.; Ma, Y.; Mai, S.; Malhado, J. P.; Malmqvist, P. Å.; Marquetand, P.; Mewes, S. A.; Norell, J.; Olivucci, M.; Oppel, M.; Phung, Q. M.; Pierloot, K.; Plasser, F.; Reiher, M.; Sand, A. M.; Schapiro, I.; Sharma, P.; Stein, C. J.; Sørensen, L. K.; Truhlar, D. G.; Ugandi, M.; Ungur, L.; Valentini, A.; Vancoillie, S.; Veryazov, V.; Weser, O.; Wesołowski, T. A.; Widmark, P.-O.; Wouters, S.; Zech, A.; Zobel, J. P.; Lindh, R. OpenMolcas: From Source Code to Insight. *J. Chem. Theory Comput.* **2019**, *15*, 5925–5964.

(12) Andersson, K.; Malmqvist, P. Å.; Roos, B. O.; Sadlej, A. J.; Wolinski, K. Second-Order Perturbation Theory with a CASSCF Reference Function. *J. Phys. Chem.* **1990**, *94*, 5483–5488.

(13) Andersson, K.; Malmqvist, P. Å.; Roos, B. O. Second-Order Perturbation Theory with a Complete Active Space Self-Consistent Field Reference Function. *J. Chem. Phys.* **1992**, *96*, 1218–1226.

(14) Roos, B. O.; Veryazov, V.; Widmark, P.-O. Relativistic Atomic Natural Orbital Type Basis Sets for the Alkaline and Alkaline-Earth Atoms Applied to the Ground-State Potentials for the Corresponding Dimers. *Theor. Chem. Acc.* **2004**, *111*, 345–351.

(15) Aquilante, F.; Pedersen, T. B.; Lindh, R. Low-Cost Evaluation of the Exchange Fock Matrix from Cholesky and Density Fitting Representations of the Electron Repulsion Integrals. *J. Chem. Phys.* **2007**, *126*, 194106.

(16) Reiher, M.; Wolf, A. Exact Decoupling of the Dirac Hamiltonian. II. The Generalized Douglas–Kroll–Hess Transformation up to Arbitrary Order. *J. Chem. Phys.* **2004**, *121*, 10945–10956.
